# The ideal candidate for the job: how epaulette sharks (*Hemiscyllium ocellatum*) have emerged as a model for climate-ready shark conservation

**DOI:** 10.1093/conphys/coag039

**Published:** 2026-06-18

**Authors:** Carmen Dobszewicz, Shamil F Debaere, Sophia M Emmons, Joel H Gayford, Aaron M Hasenei, Carolyn R Wheeler, Jodie L Rummer

**Affiliations:** School of Project Management, University of Sydney, Camperdown, Sydney, New South Wales, 2006, Australia; College of Science and Engineering, James Cook University, 1 James Cook Drive, Townsville, Queensland, 4811, Australia; College of Science and Engineering, James Cook University, 1 James Cook Drive, Townsville, Queensland, 4811, Australia; ECOSPHERE, Department of Biology, University of Antwerp, Building U, Groenenborgerlaan 171, 2020 Antwerp, Belgium; College of Science and Engineering, James Cook University, 1 James Cook Drive, Townsville, Queensland, 4811, Australia; College of Marine Science, University of South Florida, 140 7th Avenue South, MSL119, St. Petersburg, FL 33701, USA; College of Science and Engineering, James Cook University, 1 James Cook Drive, Townsville, Queensland, 4811, Australia; Shark Measurements, London, UK; College of Science and Engineering, James Cook University, 1 James Cook Drive, Townsville, Queensland, 4811, Australia; College of Science and Engineering, James Cook University, 1 James Cook Drive, Townsville, Queensland, 4811, Australia; College of Science and Engineering, James Cook University, 1 James Cook Drive, Townsville, Queensland, 4811, Australia

**Keywords:** Comparative physiology, elasmobranchs, hypoxia tolerance, ocean acidification, ocean warming, stress physiology

## Abstract

Sharks and rays are among the most threatened vertebrates; yet, mechanistic data to predict their responses to climate change remain sparse. We argue that the epaulette shark (*Hemiscyllium ocellatum*) is a tractable candidate species for conservation physiology because it is small and benthic, abundant on Great Barrier Reef flats, exceptionally tolerant of hypoxia and acidification conditions, and amenable to captive breeding and experimental work across life stages. Its distinctive appearance, accessibility and calm disposition also make it an effective flagship species for public engagement and reef conservation. A long-term breeding colony and field access have enabled experiments spanning ocean acidification, hypoxia and warming. Across studies, epaulette sharks show robust maintenance of routine behaviours in both adult and juvenile stages under elevated CO_2_, consistent with physiological traits that buffer pH changes. In contrast, warming imposes clear constraints, especially early in ontogeny. Embryos hatch earlier and smaller, aerobic scope narrows, metabolic costs rise, recovery is delayed, growth is impaired, and mortality risk increases at the upper thermal range. Adults and juveniles retain notable hypoxia tolerance, reflecting nocturnal tidepool conditions; yet, performance trade-offs may emerge when warming and low O_2_ co-occur. Together, findings so far illustrate how a resilient species can still harbour critical vulnerabilities under projected climate change conditions. Here, we outline how the epaulette shark can anchor comparative work to derive transferable thresholds (e.g. temperature–oxygen safety margins), test cross-tolerance mechanisms and validate field-relevant biomarkers for monitoring population health. Management applications follow directly, allowing habitat-specific thermal and oxygen limits for shallow flats and nursery areas to be identified, informing heatwave response and water-quality targets, and refining husbandry and welfare guidelines for research and public aquaria. While a site-attached benthic model will not capture constraints faced by pelagic elasmobranchs, leveraging this tractable flagship species to bridge mechanistic understanding and management decision points can enhance forecasts for other reef-associated chondrichthyans and support climate-ready conservation strategies.

## Introduction

Globally, climate change has profound impacts on marine environments, driving shifts in temperature, modifying water chemistry and broadly altering ecosystem dynamics (Hoegh-Guldberg & Bruno, 2010; Doney *et al.*, 2012). As such, the effects of climate change on marine environments can be considered multifaceted combining oxygen loss, acidification and ocean warming, collectively known as the ‘deadly trio’ (Bijma *et al.,* 2013). These phenomena, while individually concerning, are especially dangerous when they occur in combination, creating a highly stressed environment for marine life. Oxygen loss (hypoxia) leads to the suffocation of marine life, particularly in benthic and deeper ocean regions; ocean acidification weakens the ability of marine organisms to build and maintain their calcium carbonate structures; and ocean warming increases the rate of coral bleaching and disrupts species distribution and physiological performance (Doney *et al.,* 2009; Breitburg *et al.,* 2018; Venegas *et al.,* 2023).

Traditionally, highly sensitive species have been utilized as key indicators for each factor of the ‘deadly trio’ (algae: Gomiero *et al.,* 2018; cyanobacteria: Ribalet *et al.,* 2025; molluscs: Clements *et al.,* 2018; cyprinids: Nilsson, 2001; Cortes *et al.,* 2024). However, tolerant species offer equally valuable insights that can be used within conservation physiology. As resilient organisms that persist across a range of conditions, tolerant species offer a unique opportunity to explore the physiological mechanisms underpinning environmental resilience and serve as reliable bioindicators of ecosystem health. One such species that emerges as a viable candidate for conservation physiology and exploring marine ecosystems under a changing climate is *Hemiscyllium ocellatum* Bonnaterre, 1788 (epaulette shark), a benthic reef shark that inhabits shallow reef flats on the tropical shelf. When compared to traditionally sensitive candidate species used in conservation physiology, *H. ocellatum* situate themselves as a highly resilient species, with hard thresholds that can provide key insights into the health of coral reef ecosystems that are continuously exposed to ocean acidification, warming and oxygen loss (Wise *et al.,* 1998; Hickey *et al.,* 2012; Heinrich *et al.,* 2014; Wheeler *et al.,* 2022b see Table 1).

**Table 1:** Candidate species criteria chart indicating where *H. ocellatum* (epaulette sharks) ranks within each of the criterion, with high ranking indicating a more ideal candidate species

**Candidate species criteria**	**Low**	**Medium**	**High**
Tractability Can unique life stages of the species be maintained in a laboratory setting? Can the life cycles of the species be closed in a laboratory setting? Can wild individuals of the species be maintained in a laboratory setting?			X
Life-stage access Can unique life stages of the species be found readily in the wild? Can unique life stages be found readily in a laboratory setting?		X	
Ethics Has the species been used experimentally in the past? Can the species be released back to the wild after experimental use? Is the species ranked low by the IUCN (e.g. ‘least concern’)?		X	
Transferability Can the knowledge gain from the species be applied to other members of their class? Can the knowledge gain from the species be applied to other organisms in their environment? Can the knowledge gain from the species be applied to broader ecosystems?			X
Limits Is the species limited by one or more key climate change stressors in marine environments (ocean warming, ocean acidification, low oxygen)? Does the species demonstrate resilience to one or more key climate change stressors in marine environments (ocean warming, ocean acidification, low oxygen)? Does the species demonstrate tangible thresholds to climate change stressors?		X	

**Figure 1:**
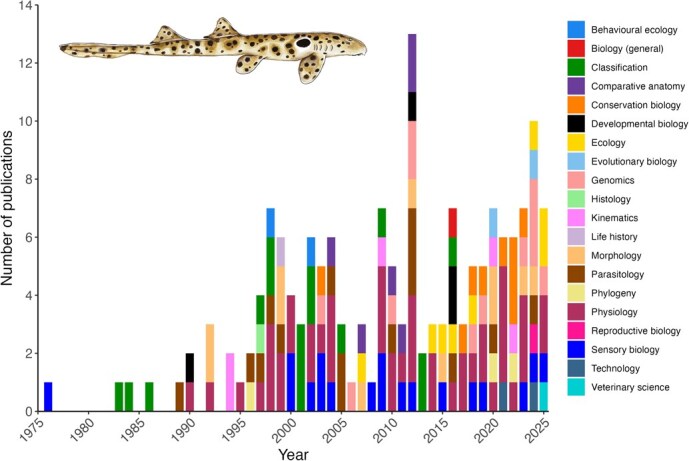
Number of publications (*N* = 163) on the epaulette shark (*H. ocellatum*) since 1975, segmented by field of research. Physiology had the greatest number of publications (*n* = 45, 27.6%), followed by sensory biology (*n* = 18, 11%) and species classification guides (‘Classification’, *n* = 16, 9.8%). The year with the greatest number of publications was 2012 (*n* = 13), spanning seven fields of study. However, 2024 had the greatest diversity, spanning eight fields of study across 10 publications. Data were compiled from publications presenting original or synthesized information on the *H. ocellatum*, identified primarily through Shark References and supplemented with Google Scholar search results. Publications that did not contribute original or synthesized data were excluded; all sources and discipline classifications are provided in the supplementary material. Illustration by Sophia Emmons*.*

To contextualize the breadth of work on *H. ocellatum*, we compiled the peer-reviewed literature in which the species was a substantive focus and assigned each paper to a primary research category; and provide the full publication list is provided in Table S1. Compared with most other elasmobranch species, *H. ocellatum* is relatively well characterized in terms of its biology, life history and ecology. This strong baseline knowledge, reflected in a large and diverse body of literature (Fig. 1), enables more complex questions and experimentation within the physiological realm (West & Carter, 1990; Heupel & Bennett, 1998, 1999, 2007; Heupel *et al.,* 1999; Payne & Rufo, 2012; Wheeler *et al.,* 2023, 2025; Lonati *et al.,* 2024; Wheeler & Rummer, 2024). As an oviparous species that breeds readily in captivity, *H. ocellatum* also enables fully controlled, cross-life-stage experimentation that is not feasible for most elasmobranchs (Heupel *et al.,* 1999; Payne & Rufo, 2012; Wheeler *et al.,* 2025).

This perspective piece aims to situate *H. ocellatum* as a strong candidate species going forward, by highlighting its robust history of physiological study, unique physiological traits and wide-scale applications. By putting forward tolerant species such as the epaulette shark, future research can expand the scope of conservation physiology to include organisms that may not display dramatic responses to environmental stressors like the ‘deadly trio’ (Bijma *et al.,* 2013) but nonetheless exhibit clear physiological thresholds. This approach offers a more comprehensive understanding of resilience and risk in changing marine ecosystems.

## Historical background

Initially described in 1788 by French naturalist Bonnaterre, it was not until the late 1990s that *H. ocellatum* was first used in physiological research. In a landmark paper, Wise *et al.* (1998) were the first to document this species’ exceptional hypoxia tolerance, followed by research documenting the conditioning effect of repeated exposure to low-oxygen conditions (Routley *et al.,* 2002) and the role of adenosine in hypoxia responses (Renshaw *et al.,* 2002). This early work had a predominantly medical focus, with these key results framed in terms of apparent parallels between the ischemic response and conditioning mechanisms of *H. ocellatum* and mammals, and the resulting implications for the treatment of stroke patients (Renshaw *et al.,* 2002). During the subsequent two decades, proteomics (Dowd *et al.,* 2010), transcriptomics (Rytkönen *et al.,* 2010) and other genetic technologies (Rytkönen *et al.,* 2012) were integrated into research on the hypoxia tolerance of *H. ocellatum*, therefore broadening the scope and impact of existing work.

**Figure 2:**
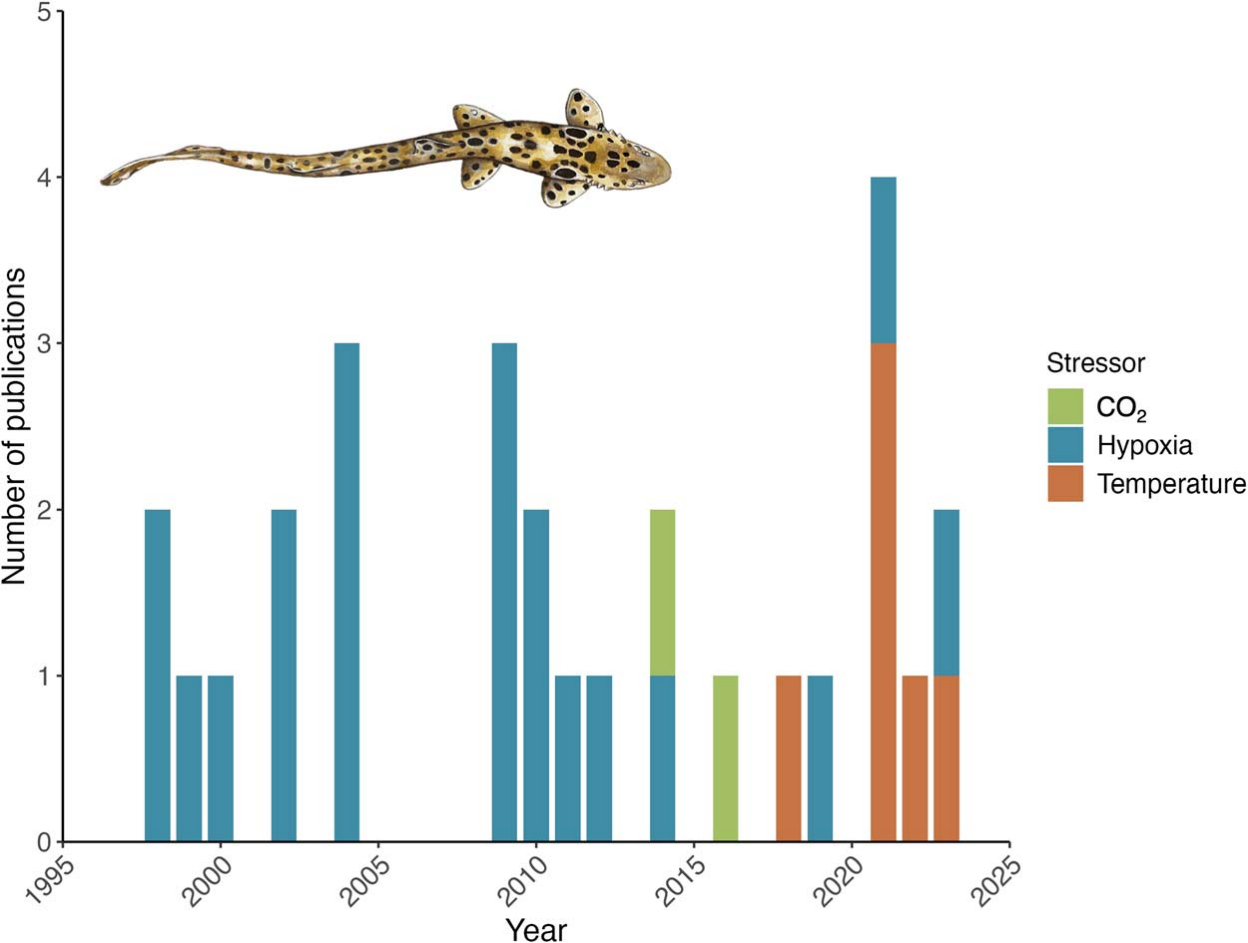
Number of studies (*N* = 28) using the epaulette shark (*H. ocellatum*) as a model species to investigate environmental stressors over time, segmented by stressor type. Studies were included if (i) the epaulette shark was a model species, (ii) an abiotic parameter was experimentally manipulated and (iii) original physiology data on *H. ocellatum* were contributed. Hypoxia was the most studied stressor (71.5%, *n* = 20), followed by temperature (21.5%, *n* = 6) and CO₂ (7%, *n* = 2). Study information and classifications can be found in supplementary material. Illustration by Sophia Emmons.

By the mid-2010s, the primary use of *H. ocellatum* had shifted. While still being utilized for medical research, studies began to use *H. ocellatum* as a model species for understanding the potential responses of marine fishes to anthropogenic climate change (Fig. 2, Table S2; Heinrich *et al.,* 2016; Gervais *et al.,* 2018; Wheeler *et al.,* 2021). The role of hypoxia and hypercapnia in driving ecological and physiological responses to climate change meant that previous work addressing the hypoxia resistance of *H. ocellatum* could be leveraged to address novel, relevant questions in the field of conservation physiology. Key milestones included the findings that *H. ocellatum* can withstand elevated levels of carbon dioxide (Heinrich *et al.,* 2014, 2016; Johnson *et al.,* 2016) but is challenged by ocean warming (Gervais et al., 2016, 2018). Finally, more recently, there has been a shift toward modelling the resilience of *H. ocellatum* in the context of potential future climate change scenarios. This shift was typified by work characterizing the thermal regime of *H. ocellatum* reared under predicted middle- and end-of-century temperatures and quantifying the resultant impacts on growth and development (Wheeler *et al.,* 2021). Together, this historical progression set the stage for focused work on the physiological traits that make *H. ocellatum* such a useful candidate species in conservation physiology.

## Unique physiological traits

### Hypoxia

The highest spring tides, and therefore some of the most severe hypoxic episodes on the reef flat, occur during the Austral summer months, which also coincides with the time period epaulette shark embryos are deposited and hatch from their egg cases (Nilsson & Renshaw, 2004). By synchronizing the timing of embryonic development with routine hypoxia, early life stages of *H. ocellatum* may precondition their tolerance to low oxygen through their heightened capacity for developmental and reversible phenotypic plasticity (Rytkönen *et al.,* 2012; Hasenei *et al.,* 2023). Preconditioning to hypoxia is primed by an increase in gene expression of oxygen and energy homeostasis regulatory pathways, including those of hypoxia-inducible factor-1 alpha, adenosine signalling components and genes affecting circulation, which are conserved among teleost fishes as well (Richards, 2009; Rytkönen *et al.,* 2012). In *H. ocellatum,* these differentially expressed genes underpin the minimization of cellular stress responses including a 50% reduction in blood pressure, bradycardia, elevated adenosine and decreased glutamate release to depress metabolism, increased protection against reactive oxygen species, and nitric oxide release for vasodilation to preserve brain function (Nilsson & Renshaw, 2004; Hickey *et al.,* 2012; Brill & Lai, 2015).

### Hypercapnia

In conjunction with nocturnal, hypoxic episodes, the reef flat is subjected to low-pH conditions similar to end-of-century ocean acidification due to peak respiration rates occurring during these periods (Routley *et al.,* 2002; Nilsson & Renshaw, 2004). As a result, pCO2 may routinely fluctuate from 188 μatm during the day to over 1000 μatm at night (Shaw *et al.,* 2012; Hannan *et al.,* 2020). *H. ocellatum* buffer pH disturbances analogously to teleost fishes by accumulating bicarbonate in the blood; however, elasmobranchs can reduce this acidosis further via branchial ammonia and urea excretion (Tresguerres *et al.,* 2010; Heinrich *et al.,* 2014). This adaptation contributes to their resilience against acid–base disturbances through increasing branchial permeability during acidosis to offload nitrogenous waste in exchange for bicarbonate loading, creating an alkaline tide that restores pH balance (Wood & Wright, 1995; Heinrich *et al.,* 2014). Additionally, *H. ocellatum* exposed to low-pH conditions elicited an increase in haemoglobin concentration, which has a greater acid–base buffering capacity than teleost haemoglobins (Heinrich *et al.,* 2014). Building on this line of physiological work, Heinrich *et al.* (2016) further observed that foraging behaviour in *H. ocellatum* is unaffected by elevated CO_2_. Ultimately, ocean acidification emerges as a climate change stressor against which *H. ocellatum* demonstrates a noted resilience, leaving ocean warming as a possible limiting factor regarding their physiological thresholds.

### Temperature

*H. ocellatum* is renowned for its hypoxia and hypercapnia compensation, but less is known regarding its thermal tolerance. Despite being exposed to highly dynamic and demanding thermal fluctuations, particularly at higher temperatures, Wheeler *et al.* (2021) found that early life stages exhibited decreased body size and metabolic performance when exposed to acute increases in temperature. Furthermore, neonate *H. ocellatum* had decreased muscle fibre density and increased satellite cells, indicating that increased temperatures impair existing muscle fibres and new fibre formation (Thomas *et al.,* 2023). Although the critical thermal maximum temperature for epaulette sharks is conserved across ontogeny (Wheeler *et al.,* 2022b), sublethal impacts from ocean warming and marine heatwaves remain ambiguous, and this species does not display evidence of behavioural thermoregulation (Gervais *et al.,* 2018). Although hypoxia and hypercapnia resilience are well documented, the extent to which these traits are maintained or compromised under simultaneous warming remains unresolved, representing a critical next step in defining multi-stressor thresholds.

### Comparative advantage

Taken together, these physiological characteristics are important not only mechanistically, but also because they shape how *H. ocellatum* functions within reef environments. Indeed, as highly variable environments, tropical coral reefs set the stage for physiological resilience across multiple stressors: temperature, hypercapnia and hypoxia. *H. ocellatum*’s hypoxia tolerance provides a distinct ecological advantage in the oxygen-variable conditions of coral reefs. During nocturnal low tides, when enclosed reef flats experience periodic hypoxia (Kinsey & Kinsey, 1967), *H. ocellatum* can remain within the reef flat matrix alongside its prey species, rather than retreating to more well-oxygenated waters. This capacity allows it to exploit resources that are unavailable to less tolerant predators. *H. ocellatum* remains active at night, when hypoxia is most likely to occur, and is known to forage during both day and night (Heupel & Bennett, 1998; Wheeler *et al.,* 2022a). The natural hyper- and hypoxic diel cycles act as hypoxia preconditioning for *H. ocellatum*, which has been shown to reduce its resting metabolic rate and critical oxygen limit for survival (Routley *et al.,* 2002; Nilsson & Renshaw, 2004). Its physiological resilience to both hypoxia and hypercapnia therefore underpins its success as a mesopredator in the coral reef environment (Heupel & Bennett, 1998).

Although *H. ocellatum* is widely regarded as the most hypoxia-tolerant elasmobranch and much of the research on this species has focused on hypoxia, its critical oxygen limits are comparable to those of other coral reef teleosts (Routley *et al.,* 2002; Nilsson & Östlund-Nilsson, 2004; Chapman & Renshaw, 2009; Rogers *et al.,* 2016; Waller *et al.,* 2024). This pattern aligns with the hypothesis that physiological traits evolve in response to the prevailing oxygen conditions of a species’ environment, with critical oxygen limits reflecting local *P*O_2_ regimes (Seibel & Deutsch, 2020). The mechanisms by which *H. ocellatum* endures acute hypoxia (e.g. bradycardia and metabolic suppression) are unique among elasmobranchs (Nilsson & Renshaw, 2004; Stensløkken *et al.,* 2004), yet may parallel strategies employed by other coral reef vertebrates adapted to oxygen-variable habitats. When full aerobic scope is considered, however, *H. ocellatum*’s metabolic performance becomes constrained at oxygen levels below full air saturation (Emmons, personal communication). Thus, while it exhibits distinctive physiological responses to hypoxia, its overall aerobic tolerance reflects adaptation to the same environmental pressures shaping other coral reef species.

### Broader relevance

Beyond its ecological success on reef flats, *H. ocellatum* also offers broader comparative value as a model for understanding tolerance limits in other reef-associated vertebrates. Indeed, *H. ocellatum* represents an excellent model species for other coral reef vertebrates, particularly in climate change contexts. While its physiological coping strategies are unique, it displays similar environmental tolerance limits that can be used to understand changes in performance under future climate scenarios. For example, *H. ocellatum* is vulnerable to rising temperatures with a critical thermal maximum around 36°C conserved across juvenile to adult life stages, sex and body size (Wheeler *et al.,* 2022b). Similarly, tropical damselfish species *Acanthochromis polyacanthus* has a critical thermal maximum of 36.6°C, which is dependent on individual size and life stage (Clark *et al.,* 2017). Alarmingly, these temperatures have already been observed in the wild (AIMS, 2022).

However, despite its value as a model species for other coral reef vertebrates, *H. ocellatum* may be particularly vulnerable to climate change. As a long-lived species with low fecundity relative to teleosts and low mutation rates, it relies heavily on physiological plasticity to cope with environmental variation influencing exposure to hypoxic conditions, with the speculation that this phenotypic plasticity might assist in coping with ocean warming as well (Chin *et al.,* 2010; Rummer *et al.,* 2022; Hasenei *et al.,* 2023; Sendell-Price *et al.,* 2023). In this regard, phenotypic plasticity over generational adaptation is the best chance for site-attached, long-lived species like *H. ocellatum* to keep pace with the accelerating rate of environmental change.

## Applications in conservation physiology and management

### Comparative and mechanistic research

Testing the synergetic effects of hypoxia, hypercapnia and warming in *H. ocellatum* provides critical insight into how multiple climate stressors interact to shape physiological performance (Wise *et al.,* 1998; Pörtner and Peck, 2010; Rytkönen *et al.,* 2012; Heinrich *et al.,* 2014; Wheeler *et al.,* 2022b). By examining metabolic changes, cardiac regulation and behavioural activity under combined stress conditions, researchers can identify thresholds that are not apparent when stressors are studied in isolation. These integrative experiments reveal the extent to which resilience mechanisms are additive, antagonistic or synergistic, offering a more realistic picture of how reef-associated elasmobranchs will fare in future environments. While interpreting these responses, it is important to recognize that some aspects of *H. ocellatum* biology, such as its benthic, site-attached ecology, small body size and routine nocturnal exposure to tide-pool hypoxia, are not broadly representative of more mobile or pelagic species. Nonetheless, establishing these interaction effects is essential for predicting ecosystem-level consequences, as predator performance under multi-stress scenarios directly influences prey dynamics and energy flow in coral reef systems.

Determining temperature and oxygen safety margins in *H. ocellatum* provides a mechanistic foundation for assessing resilience across reef-associated elasmobranchs. By quantifying thresholds such as the critical oxygen tension, aerobic scope and upper thermal limits, researchers can establish metrics that are transferable to species occupying similar ecological niches. At the same time, some traits such as oviparity, low fecundity relative to teleosts and metabolic scaling associated with small body size limit direct extrapolation across taxa with different life histories or energetic strategies. Comparative analyses of these safety margins could reveal whether tolerance in *H. ocellatum* represents a unique adaptation or reflects broader patterns among reef predators. Such generalizable benchmarks are essential for predicting species vulnerability under climate change, even if not all characteristics are transferable, and for guiding conservation strategies that prioritize physiological resilience in oxygen-variable reef habitats.

Positioning *H. ocellatum* as a model species enables direct linkage between physiological tolerance and ecosystem-level processes. Its well-characterized responses to hypoxia, hypercapnia and warming provide mechanistic insights into how predator performance shapes prey availability, habitat use and trophic interactions in coral reef systems. Although some features of its biology, such as its reliance on shallow, structurally complex reef flats and its predictable exposure to low-oxygen microhabitats, are not shared by all reef predators, these distinctions do not detract from its value as a model for understanding cross-tolerance mechanisms. By integrating physiological thresholds with ecological observations, researchers can predict how shifts in individual resilience cascade into altered community dynamics and energy flow. This model framework not only advances comparative physiology but also informs conservation strategies by connecting organismal function to the broader resilience of reef ecosystems under climate change. As such, *H. ocellatum* provides an essential benchmark for testing cross-tolerance mechanisms—particularly whether hypoxia resilience persists, weakens or interacts antagonistically with thermal stress—and for calibrating comparative thresholds across other site-attached reef sharks and rays.

### Field-ready toolbox for tractability and longitudinal monitoring

Minimally invasive, field-adapted sampling methods make *H. ocellatum* especially tractable for paired lab–field studies and longitudinal monitoring. Rapid point-of-care blood assays provide immediate, interpretable proxies for oxygen-carrying capacity and acute metabolic load: haemoglobin (HemoCue®) as an O₂-carrying proxy; handheld measures of glucose, lactate and beta-hydroxybutyrate/ketones; plus haematocrit and, where logistics permit, basic acid–base and ion panels (Schwieterman *et al.,* 2019; Wheeler *et al.,* 2025). Validated short-term storage protocols allow aliquots to be frozen for expanded hormone, metabolite or biochemical panels in the laboratory while preserving field triage capability (Heupel *et al.,* 1999; Schwieterman *et al.,* 2019; Wheeler *et al.,* 2025).

Animal handling artefact tests indicate red blood cell stability after brief air exposure or exhaustive exercise across multiple elasmobranchs, supporting the reliability of routine haematology under typical field conditions (Schwieterman *et al.,* 2021). Complementing these blood measures is a validated, non-lethal liver biopsy, which permits repeated sampling for transcriptomics and biochemistry (RNA-seq, glycogen/lipids, oxidative enzymes), therefore enabling within-individual, time-series inference on energetic regulation and stress responses (Hasenei *et al.,* 2025). Furthermore, preserving tissue in RNAlater or snap-freezing in liquid N₂ while minimizing handling time secures high RNA integrity for longitudinal ‘omics’. Given the accessibility of the sequenced and well-annotated genome of *H. ocellatum* (Sendell-Price, 2023), this presents a rare opportunity in elasmobranch conservation genetics to characterize inter- and intraspecific molecular adaptation and acclimation responses to environmental stressors. Together, these methods deliver a practical, interpretable toolkit that allows for rapid field triage during heat or hypoxia events, paired physiological baselines tied to temperature–oxygen safety margins, and longitudinal mechanistic data linking energy state and gene expression to performance thresholds. Applied management uses include heatwave triage to flag at-risk populations or flats, identification of nursery or foraging areas approaching critical limits, and evidence-based welfare and husbandry protocols using serial blood sampling and non-lethal tissue sampling to inform care and intervention.

### Educational and conservation applications

Beyond research and monitoring, the tractability of *H. ocellatum* also creates opportunities for public engagement and conservation communication. Its small size, distinctive markings and observable walking behaviour (Dudgeon *et al.*, 2020; Porter *et al.,* 2022) make this species an ideal ambassador for translating physiological science into engaging public narratives. Public aquaria can leverage live *H. ocellatum* in interpretive displays and hands-on modules that pair short demonstrations with real-time temperature and oxygen data for use in school programmes and curator talks to show how field assays inform conservation action. Framing climate impacts around a single relatable species simplifies complex messages, connecting thresholds for hypoxia and warming to tangible outcomes such as reduced foraging success or nursery loss. This narrative approach both motivates protective behaviours and provides a clear conduit for scientists and managers to communicate urgent, locally relevant conservation measures.

By quantifying temperature–oxygen safety margins and validating field-ready biomarkers, research on *H. ocellatum* supports management actions such as identifying nursery and foraging sites at risk during heatwaves or hypoxic events; informing thresholds for water-quality targets; developing triage frameworks for localized die-offs; and refining husbandry protocols for aquaria and conservation breeding programmes. These applications translate mechanistic tolerance metrics into actionable decision tools for managers overseeing climate-exposed shallow reef systems.

### Ethical considerations

The utilization of candidate species for research in conservation physiology highlights the need for explicit consideration of ethical and welfare implications, particularly given the frequent use of captive holding, experimental manipulation, and repeated sampling. While the use of a model species can reduce direct research pressure on threatened organisms, ethical responsibility extends beyond species selection to encompass sample size, animal welfare and experimental intensity (Tannenbaum & Bennett, 2015). Captive and repeatedly sampled individuals may experience cumulative stress that can affect both welfare and physiological validity, underscoring the importance of discerning husbandry, minimally invasive techniques and transparent justification of sampling techniques (Minteer & Collins, 2013; Fischer & Romero, 2019; Beaulieu, 2024). Importantly, the conservation value of candidate species research rests on a clear rationale, whereby impacts on study organisms are justified by anticipated gains in conservation or management. Mindful attention towards ethical frameworks and welfare considerations strengthens both the scientific robustness and societal legitimacy of the utilization of candidate species approaches in conservation contexts.

## Future directions

With these practical and ethical considerations in mind, several priority directions emerge for future work with *H. ocellatum*. Several biomarkers have been physiologically validated for use with *H.ocellatum*, as discussed throughout the previous sections (e.g. blood HCO_3_, circulating adenosine/glutamate and haemoglobin concentrations). Building upon this physiological knowledge, logical next steps will be to biologically validate easily quantifiable indicators in wild, free-ranging sharks, allowing for the documentation of responses to environmental change (Madliger *et al.,* 2018). Imperative to testing the biological validity and ecological relevance of these metrics will be to establish baseline values across life stages, seasons, sex and reproductive status (Dantzer *et al.,* 2014). In that regard, the smaller size of *H. ocellatum*, relative to many other reef shark species, allows for rapid blood sampling upon capture, thereby minimizing exogenous effects of capture and handling when collecting reference samples (Romero & Reed, 2005; Dantzer *et al.,* 2014; Lawrence *et al.,* 2018). Furthermore, the availability of a fully sequenced and annotated genome renders *H. ocellatum* an exceptionally tractable model for high-resolution ‘omics’ research (Sendell-Price *et al.,* 2023). Unlike many elasmobranch species characterized by massive, repetitive and unmapped genomes, *H. ocellatum* provides a clear genetic template that allows for precise transcriptomic mapping and functional gene annotation.

Following this biological validation and establishing baseline references, an atlas incorporating these baseline values across populations will be pivotal in monitoring, informing and refining case-specific management efforts for *H. ocellatum* throughout their range (Cooke *et al.,* 2013). Over a short timescale, such an atlas can help assess susceptibility to abrupt climatic events (e.g. effects of marine heatwaves, hypoxia following severe rainfall), loss of habitat complexity and refuge following storms, etc. Over a longer timescale, these physiological biomarkers will help shed light on the prolonged effects of global change, how *H. ocellatum* copes with (and potentially adapts to) these environmental challenges and helps predict ecosystem responses to future ocean conditions. Additionally, although *H. ocellatum* is a relatively well-studied shark species, accurate physiological data are lacking for many other species of the genus *Hemiscyllium* (Müller and Henle, 1838). It will therefore be worthwhile to assess the extent to which our knowledge from *H. ocellatum* can be applied to other closely related *Hemiscyllium* species of conservation concern throughout the Western South Pacific into Papua New Guinea (VanderWright *et al.*, 2022).

Aside from the primarily haematological biomarkers considered throughout this perspective, a knowledge gap also remains as to how the mechanistic insights gained thus far translate to behavioural responses to environmental change. Behavioural actions often easily connect physiological data with practical conservation and management outcomes (Cooke *et al.,* 2014), and a wide range of behavioural tools are available to study these actions (e.g. biotelemetry and biologging tools). However, behavioural studies on *H. ocellatum* are limited. In one study, Gervais *et al.* (2018) reported no apparent behavioural thermoregulation in the species; yet, anecdotal evidence has demonstrated *H. ocellatum* moving between tidepools. However, whether this movement is an avoidance behaviour (i.e. in order to avoid certain conditions in one tidepool) remains speculative. Such movement behaviours are easily quantifiable using minimally invasive techniques and changes in their occurrence could indicate responses to challenging environments (Cooke *et al.,* 2014; Gervais *et al.,* 2018; Nay *et al.,* 2021).

As implied throughout this perspective, *H. ocellatum* is often considered to be a relatively stress-tolerant elasmobranch species, being able to withstand low-oxygen environments and associated acid–base disturbances. However, stressors come in many shapes and sizes, and the elasmobranch stress axis (the neuroendocrine hypothalamus–pituitary–interrenal axis) is poorly understood (Anderson, 2012; Bouyoucos *et al.,* 2021; Schoen *et al.,* 2024). Whether *H. ocellatum* exhibits comparable tolerance to additional climate stressors beyond hypoxia and hypercapnia remains unclear. The well-documented developmental stages of this species and its ability to successfully reproduce in captivity make it a suitable candidate for exploring some of these unanswered questions in stress physiology, including the development of the stress axis, transgenerational effects of stressor exposure (acute or chronic), and habituation or acclimation to repeated or sustained stressors.

## Conclusions

This perspective piece aimed to consolidate over two decades of physiological research utilizing *H. ocellatum,* from early experiments situated in medical research to more recent studies investigating climate change stressors in marine environments. Fundamental research has highlighted the impressive tolerance of this species to hypoxia and hypercapnia, while also identifying ocean warming as a key limiting factor. With this knowledge in mind, we highlighted broader applications of research on *H. ocellatum,* particularly for comparative studies and approaches that can be integrated across field and laboratory settings. At the same time, the limitations of *H. ocellatum* must be recognized when considering exactly how broadly inferences from this species can be applied. Being clear about both its strengths and its limitations is essential for understanding how *H. ocellatum* can be positioned as a candidate species for conservation physiology.

Taken together, the existing literature positions *H. ocellatum* as a tractable and informative model for conservation physiology, particularly for identifying physiological thresholds, ontogenetic bottlenecks, and field-relevant indicators of climate vulnerability. More broadly, this perspective supports the value of resilient candidate species, not only sensitive ones, for defining physiologically meaningful thresholds under the major stressors facing marine systems globally. We therefore see *H. ocellatum* as one important component of a wider portfolio of model marine species spanning habitats, life histories and adaptive strategies. Building such a portfolio will strengthen comparative research and improve the conservation relevance of mechanistic studies under rapid environmental change.

## Supplementary Material

Web_Material_coag039

## Data Availability

No data were generated or analysed during this work.
