# Supplementary material for: The ideal candidate for the job: how epaulette sharks (Hemiscyllium ocellatum) have emerged as a model for climate-ready shark conservation
Source: Conserv Physiol. 2026 Jun 18;14(1):coag039. doi: 10.1093/conphys/coag039 (PMC13278842; doi:10.1093/conphys/coag039)
Supplement: Web_Material_coag039 [file web_material_coag039.zip › SI_figure references with title and authors.pdf]

The ideal candidate for the job: How epaulette sharks (*Hemiscyllium ocellatum*) have emerged as a model for climate-ready shark conservation

Carmen Dobszewicz [1,2], Shamil F. Debaere [2,3], Sophia M. Emmons [2,4], Joel H. Gayford [2,5], Aaron Hasenei [2], Carolyn Wheeler [2], Jodie L. Rummer [2]

[1] School of Project Management, University of Sydney, Sydney, New South Wales, Australia
[2] College of Science and Engineering, James Cook University, Townsville, Queensland, Australia
[3] ECOSPHERE, Department of Biology, University of Antwerp, Antwerp, Belgium
[4] College of Marine Science, University of South Florida, St. Petersburg, FL, USA
[5] Shark Measurements, London, UK

Supplemental information: sources used in Figures 1 and 2

Figure 1

Allen, G.R., Erdmann, M.V., White, W.T., Fahmi, Dudgeon, C.L., 2016. Review of the bamboo shark genus Hemiscyllium (Orectolobiformes: Hemiscyllidae). Journal of the Ocean Science Foundation, 23, 51–97.

Alves, L.M.F., Lemos, M.F.L., Cabral, H., Novais, S.C., 2022. Elasmobranchs as bioindicators of pollution in the marine environment. Marine Pollution Bulletin 176, 113418. https://doi.org/10.1016/j.marpolbul.2022.113418

Appleby, M., Raoult, V., Broadhurst, M.K., Gaston, T., 2024. Can denticle morphology help identify southeastern Australian elasmobranchs? Journal of Fish Biology 104, 1848–1859. https://doi.org/10.1111/jfb.15704

Ashton, K., Pritchard, G., Renshaw, G. M. C., Headrick, J. P., 2006. Fish and chips: Cardiac gene expression profiling in the anoxia tolerant epaulette shark, Journal of Molecular and Cellular Cardiology, 41733.

Atkinson, C.J.L. & Collin, S.P., 2012. Structure and Topographic Distribution of Oral Denticles in Elasmobranch Fishes. Biological Bulletin, 222(1), 26–34.

Atkinson, C.J.L., Collin, S.P., 2025. Surface morphology and distribution of oropharyngeal taste papillae in sharks and rays (Elasmobranchii, Chondrichthyes): Implications for gustatory sensitivity. Journal of Anatomy 247, 924–952. https://doi.org/10.1111/joa.14278

Avrit, S., 2024. An Economic and Ecological Gap Analysis of Epaulette Sharks, Hemiscyllium. California State University, Long Beach.

Baldwin, J., Wells, R.M.G., 1990. Oxygen transport potential in tropical elasmobranchs from the Great Barrier Reef: relationship between haematology and blood viscosity. Journal of Experimental Marine Biology and Ecology 144, 145–155. https://doi.org/10.1016/0022-0981(90)90025-8

Becerril-García, E.E., Arellano-Martínez, M., Bernot-Simon, D., Hoyos-Padilla, E.M., Galván-Magaña, F., Godard-Codding, C., 2020. Steroid hormones and chondrichthyan reproduction: physiological functions, scientific research, and implications for conservation. PeerJ 8, e9686. https://doi.org/10.7717/peerj.9686

Bennett, M.B., Heupel, M.R., Bennett, S.M., Parker, A.R., 1997. Sheina orri (Myodocopa: Cypridinidae), an ostracod parasitic on the gills of the epaulette shark, Hemiscyllium ocellatum (Elasmobranchii: Hemiscyllidae). International Journal for Parasitology 27, 275–281. https://doi.org/10.1016/S0020-7519(96)00201-9

Bouyoucos, I.A., 2021. Walking sharks cannot beat the heat. Conservation Physiology 9, coab035. https://doi.org/10.1093/conphys/coab035

Bouyoucos, I.A., Simpfendorfer, C.A., Rummer, J.L., 2019. Estimating oxygen uptake rates to understand stress in sharks and rays. Rev Fish Biol Fisheries 29, 297–311. https://doi.org/10.1007/s11160-019-09553-3

Bozzano, A., Collin, S.P., 2000. Retinal Ganglion Cell Topography in Elasmobranchs. Brain Behavior and Evolution 55, 191–208. https://doi.org/10.1159/000006652

Buckley, K.A., Crook, D.A., Pillans, R.D., Smith, L., Kyne, P.M., 2018. Sustainability of threatened species displayed in public aquaria, with a case study of Australian sharks and rays. Reviews in Fish Biology and Fisheries 28, 137–151. https://doi.org/10.1007/s11160-017-9501-2

Burreson, E., 1989. Hematozoa of Fishes From Heron Island, Australia, With the Description of 2 New Species of Trypanosoma. Australian Journal of Zoology 37, 15–23. https://doi.org/10.1071/ZO9890015

Carpenter, K.E. & Niem, V.H., 1998. FAO species identification guide for fishery purposes. The living marine resources of the Western Central Pacific. Volume 2. Cephalopods, crustaceans, holothurians and sharks. Rome, FAO. 1998: 687–1396.

Cavanagh, R.D., Kyne, P.M., Fowler, S.L., Musick, J.A., Bennett, M.B., 2003. The Conservation Status of Australasian Chondrichthyans. Report of the IUCN Shark Specialist Group Australia and Oceania Regional Red List Workshop. The University of Queensland, School of Biomedical Sciences, Brisbane, Australia.

Ceccarelli, D.M., Frisch, A.J., Graham, N.A.J., Ayling, A.M., Beger, M., 2014. Habitat partitioning and vulnerability of sharks in the Great Barrier Reef Marine Park. Reviews in Fish Biology and Fisheries 24, 169–197. https://doi.org/10.1007/s11160-013-9324-8

Chapman, C. A., 2009. Anoxia Survival Strategies in the Grey Carpet Shark (Chiloscyllium punctatum) and the Epaulette Shark (Hemiscyllium ocellatum). PhD Thesis, School of Physiotherapy and Exercise Science, Griffith University. https://doi.org/10.25904/1912/594.

Chapman, C.A., Harahush, B.K., Renshaw, G.M.C., 2011. The physiological tolerance of the grey carpet shark (Chiloscyllium punctatum) and the epaulette shark (Hemiscyllium ocellatum) to anoxic exposure at three seasonal temperatures. Fish Physiol Biochem 37, 387–399. https://doi.org/10.1007/s10695-010-9439-y

Chapman, C.A., Renshaw, G.M.C., 2009. Hematological responses of the grey carpet shark (Chiloscyllium punctatum) and the epaulette shark (Hemiscyllium ocellatum) to anoxia and re-oxygenation. Journal of Experimental Zoology Part A: Ecological Genetics and Physiology 311A, 422–438. https://doi.org/10.1002/jez.539

Compagno, L.J.V., 1984. FAO Species Catalogue. Vol 4: Sharks of the world, Part 1 - Hexanchiformes to Lamniformes. FAO Fisheries Synopsis No. 125, 4(1): 1–250.

Compagno, L.J.V., 2001. FAO Species Catalogue. Vol 2: Sharks of the world, Bullhead, mackerel and carpet sharks (Heterodontiformes, Lamniformes and Orectolobiformes). FAO Species Catalogue for Fishery Purposes No. 1, Vol. 2: 1–250.

Compagno, L.J.V., 2005. Checklist of living Chondrichthyes. In W.C. Hamlett (Ed.), Reproductive biology and phylogeny of chondrichthyes: sharks, rays and chimaeras, Vol. 3 (pp. 503–548). Science Publishers, Endfield, USA.

Compagno, L.J.V. & Niem, V.H., 2005. Part Sharks: Hexanchidae, Echinorhinidae, Squalidae, Squatinidae, Heterodontidae, Parascyllidae, Bracheluridae, Orectolobidae, Hemiscyllidae, Odontaspididae, Scyliorhinidae, Proscyllidae, Triakidae, In K.E. Carpenter and V.H. Niem(eds.), FAO Identification Guide for Fishery Purposes. The Living Marine Resources of the Western Central Pacific. Food and Agriculture Organization, Rome: 1195–1232, 1235–1259, 1264–1267, 1279–1295, 1297–1304, 1312–1360.

da Silva, J.P.C.B., De Carvalho, M.R., 2015. Morphology and phylogenetic significance of the pectoral articular region in elasmobranchs (Chondrichthyes). Zoological Journal of the Linnean Society 175, 525–568. https://doi.org/10.1111/zoj.12287

da Silva, J.P.C.B., Vaz, D.F.B., 2023. Morphology and phylogenetic significance of the pelvic articular region in elasmobranchs (Chondrichthyes). Cladistics 39, 155–197. https://doi.org/10.1111/cla.12528

de Carvalho, M.R., 1996. Higher-Level Elasmobranch phylogeny, basal Squaleans, and paraphyly. In Stiassny, M.L.J., Parenti, L.R., & Johnson, G.D.(ed.), Interrelation of Fishes 3. Academic Press, New York. 35–62 pp.

Devaux, J.B.L., Hickey, A.J.R., Renshaw, G.M.C., 2023. Succinate-mediated reactive oxygen species production in the anoxia-tolerant epaulette (Hemiscyllium ocellatum) and grey carpet (Chiloscyllium punctatum) sharks. Biology Letters 19, 20230344. https://doi.org/10.1098/rsbl.2023.0344

Devaux, J.B.L., Hickey, A.J.R., Renshaw, G.M.C., 2019. Mitochondrial plasticity in the cerebellum of two anoxia-tolerant sharks: contrasting responses to anoxia/re-oxygenation. Journal of Experimental Biology 222, jeb191353. https://doi.org/10.1242/jeb.191353

Dingerkus, G. & de Fino, T.C., 1983. A revision of the orectolobiform shark family Hemiscyllidae (Chondrichthyes, Selachii). Bulletin of the American Museum of Natural History, 176(1), 1–93.

Dowd, W.W., Renshaw, G.M.C., Cech, J.J., Kültz, D., 2010. Compensatory proteome adjustments imply tissue-specific structural and metabolic reorganization following episodic hypoxia or anoxia in the epaulette shark (Hemiscyllium ocellatum). Physiological Genomics 42, 93–114. https://doi.org/10.1152/physiolgenomics.00176.2009

Dudgeon, C.L., Corrigan, S., Yang, L., Allen, G.R., Erdmann, M.V., Fahmi, Sugeha, H.Y., White, W.T., Naylor, G.J.P., 2020. Walking, swimming or hitching a ride? Phylogenetics and biogeography of the walking shark genus Hemiscyllium. Marine & Freshwater Research 71, 1107–1117. https://doi.org/10.1071/MF19163

Ebert, D.A, Fowler, S., Compagno, L.J.V., 2013. Sharks of the World – A fully illustrated guide. Wild Nature Press, ISBN 978–0–9573946–0–5: 528pp.

Eiras, J.C., Lu, Y.S., Gibson, D.I., Fiala, I., Saraiva, A., Cruz, C., Santos, M.J., 2012. Synopsis of the species of Chloromyxum Mingazinni, 1890 (Myxozoa: Myxosporea: Chloromyxidae). Systematic Parasitology 83, 203–225. https://doi.org/10.1007/s11230-012-9380-9

Ermakova, G.V., Meyntser, I.V., Zaraisky, A.G., Bayramov, A.V., 2024. Loss of noggin1, a classic embryonic inducer gene, in elasmobranchs. Scientific Reports 14, 3805. https://doi.org/10.1038/s41598-024-54435-9

Fahmi, Putra, Y., Oktaviyani, S., Jutan, Y., Haris, A.S., Jamlaay, F., 2025. Ecology, conservation, and future perspective of walking sharks (Hemiscyllium spp.) in Indonesia. Pacific Conservation Biology 31, PC24088. https://doi.org/10.1071/PC24088

Fuery, C.J., Attwood, P.V., Withers, P.C., Yancey, P.H., Baldwin, J., Guppy, M., 1997. Effects of Urea on M4-Lactate Dehydrogenase from Elasmobranchs and Urea-Accumulating Australian Desert Frogs. Comparative Biochemistry and Physiology Part B: Biochemistry and Molecular Biology 117, 143–150. https://doi.org/10.1016/S0305-0491(96)00287-8

Gauthier, A.R.G., Whitehead, D.L., Tibbetts, I.R., Bennett, M.B., 2019. Comparative morphology of the electrosensory system of the epaulette shark Hemiscyllium ocellatum and brown-banded bamboo shark Chiloscyllium punctatum. Journal of Fish Biology 94, 313–319. https://doi.org/10.1111/jfb.13893

Gayford, J.H., Brazeau, M.D., Naylor, G.J.P., 2024. Evolutionary trends in the elasmobranch neurocranium. Scientific Reports 14, 11471. https://doi.org/10.1038/s41598-024-62004-3

Gayford, J.H., Rummer, J.L., 2025. Tonic immobility in cartilaginous fishes (Chondrichthyes): function, evolutionary history, and future directions. Reviews in Fish Biology and Fisheries 35, 1301–1315. https://doi.org/10.1007/s11160-025-09958-3

Gervais, C., Mourier, J., Rummer, J.L., 2016. Developing in warm water: irregular colouration and patterns of a neonate elasmobranch. Marine Biodiversity 46, 743–744. https://doi.org/10.1007/s12526-015-0429-2
Gervais, C.R., Nay, T.J., Renshaw, G., Johansen, J.L., Steffensen, J.F., Rummer, J.L., 2018. Too hot to handle? Using movement to alleviate effects of elevated temperatures in a benthic elasmobranch, Hemiscyllium ocellatum. Mar Biol 165, 162. https://doi.org/10.1007/s00227-018-3427-7
Gleeson, R.J., Adlard, R.D., 2012. Phylogenetic relationships amongst Chloromyxum Mingazzini, 1890 (Myxozoa: Myxosporea), and the description of six novel species from Australian elasmobranchs. Parasitology International 61, 267–274. https://doi.org/10.1016/j.parint.2011.10.008
Gleeson, R.J., Bennet, M.B., Adlard, R.D., 2010. First taxonomic description of multivalvulidan myxosporean parasites from elasmobranchs: Kudoa hemiscylli n.sp. and Kudoa carcharhini n.sp. (Myxosporea: Multivalvulidae). Parasitology 137, 1885–1898. https://doi.org/10.1017/S0031182010000855
Goto, T., Nishida, K., Nakaya, K., 1999. Internal morphology and function of paired fins in the epaulette shark,hemiscyllium ocellatum. Ichthyological Research 46, 281–287. https://doi.org/10.1007/BF02678514
Goto, T., 2001. Comparative Anatomy, Phylogeny and Cladistic Classification of the order Orectolobiformes (Chondrichthyes, Elasmobranchii). Memoirs of the Graduate School of Fisheries Sciences, Hokkaido University, 48, 1–100.
Grew, M., Raoult, V., Gaston, T.F., 2024. Behavioural response of benthic elasmobranchs to a neodymium magnet under controlled laboratory conditions. Fisheries Research 271, 106926. https://doi.org/10.1016/j.fishres.2023.106926
Guinot, G., Cavin, L., 2020. Distinct Responses of Elasmobranchs and Ray-Finned Fishes to Long-Term Global Change. Frontiers in Ecology and Evolution Volume 7-2019. https://doi.org/10.3389/fevo.2019.00513
Hardie, D.C., Hebert, P.D.N., 2003. The nucleotypic effects of cellular DNA content in cartilaginous and ray-finned fishes. Genome 46, 683–706. https://doi.org/10.1139/g03-040
Hart, N.S., Theiss, S.M., Harahush, B.K., Collin, S.P., 2011. Microspectrophotometric evidence for cone monochromacy in sharks. Naturwissenschaften 98, 193–201. https://doi.org/10.1007/s00114-010-0758-8
Hasenei, A., Foyle, L., Rummer, J., 2025. A novel, nonlethal liver biopsy procedure in an elasmobranch. Australian Veterinary Journal 103, 407–410. https://doi.org/10.1111/avj.13432
Heinrich, D.D.U., Rummer, J.L., Morash, A.J., Watson, S.-A., Simpfendorfer, C.A., Heupel, M.R., Munday, P.L., 2014. A product of its environment: the epaulette shark (Hemiscyllium ocellatum) exhibits physiological tolerance to elevated environmental CO(2). Conservation Physiology 2, cou047. https://doi.org/10.1093/conphys/cou047
Heinrich, D.D.U., Watson, S.-A., Rummer, J.L., Brandl, S.J., Simpfendorfer, C.A., Heupel, M.R., Munday, P.L., 2016. Foraging behaviour of the epaulette shark Hemiscyllium ocellatum is not affected by elevated CO2. ICES Journal of Marine Science 73, 633–640. https://doi.org/10.1093/icesjms/fsv085
Herman, J., Hovestadt-Euler, M., Hovestadt, D.C., 1992. Contributions to the study of the comparative morphology of teeth and other relevant ichthyodorulites in living superspecific taxa of Chondrichthyan fishes. Part A: Selachii. No. 4: Order: Orectolobiformes Families: Brachaeluridae, Giglymostomatidae, Hemiscylliidae, Orectolobidae, Parascylliidae,

Rhiniodontidae, Stegostomatidae. Order: Pristiophoriformes - Family: Pristiophoridae Order: Squatiniformes - Family: Squatinidae. Bulletin de l'Institut Royal des Sciences Naturelles de Belgique, Biologie, 62, 193–254.
Heupel, M. R., 1999. Life history of the epaulette shark Hemiscyllium ocellatum on Heron Island Reef, Great Barrier Reef, Australia, with comments on other reef sharks. PhD Thesis, School of Biomedical Sciences, The University of Queensland. https://doi.org/10.14264/734685.
Heupel, M.R., Bennett, M.B., 2007. Estimating Abundance of Reef-Dwelling Sharks: A Case Study of the Epaulette Shark, Hemiscyllium ocellatum (Elasmobranchii: Hemiscyllidae)1. pasc 61, 383–394. https://doi.org/10.2984/1534-6188(2007)61%255B383:EAORSA%255D2.0.CO;2
Heupel, M.R., Bennett, M.B., 1999. The occurrence, distribution and pathology associated with gnathiid isopod larvae infecting the epaulette shark, Hemiscyllium ocellatum. International Journal for Parasitology 29, 321–330. https://doi.org/10.1016/S0020-7519(98)00218-5
Heupel, M.R., Bennett, M.B., 1998a. Observations on the diet and feeding habits of the epaulette shark, Hemiscyllium ocellatum (Bonnaterre), on Heron Island Reef, Great Barrier Reef, Australia. Mar. Freshwater Res. 49, 753–756. https://doi.org/10.1071/mf97026
Heupel, M.R., Bennett, M.B., 1998b. Infection of the epaulette shark, Hemiscyllium ocellatum (Bonnaterre), by the nematode parasite Proleptus australis Bayliss (Spirurida: Physalopteridae). Journal of Fish Diseases 21, 407–414. https://doi.org/10.1046/j.1365-2761.1998.00121.x
Heupel, M.R., Bennett, M.B., 1997. Histology of dart tag insertion sites in the epaulette shark. Journal of Fish Biology 50, 1034–1041. https://doi.org/10.1111/j.1095-8649.1997.tb01628.x
Heupel, M.R., Bennett, M.B., 1996. A myxosporean parasite (Myxosporea: Multivalvulida) in the skeletal muscle of epaulette sharks, Hemiscyllium ocellatum (Bonnaterre), from the Great Barrier Reef. Journal of Fish Diseases 19, 189–191. https://doi.org/10.1111/j.1365-2761.1996.tb00700.x
Heupel, M. R., Simpfendorfer, C.A., Bennett, M.B., 1999. Skeletal deformities in elasmobranchs from Australian waters. Journal of Fish Biology 54, 1111–1115. https://doi.org/10.1111/j.1095-8649.1999.tb00861.x
Heupel, M.R., Whittier, J.M., Bennett, M.B., 1999. Plasma steroid hormone profiles and reproductive biology of the epaulette shark, Hemiscyllium ocellatum. Journal of Experimental Zoology 284, 586–594. https://doi.org/10.1002/(SICI)1097-010X(19991001)284:5%253C586::AID-JEZ14%253E3.0.CO;2-B
Hickey, A.J.R., Renshaw, G.M.C., Speers-Roesch, B., Richards, J.G., Wang, Y., Farrell, A.P., Brauner, C.J., 2012. A radical approach to beating hypoxia: depressed free radical release from heart fibres of the hypoxia-tolerant epaulette shark (Hemiscyllum ocellatum). J Comp Physiol B 182, 91–100. https://doi.org/10.1007/s00360-011-0599-6
Hutchins, J.B., 2001. Checklist of the fishes of Western Australia. Records of the Western Australian Museum, Supplement 63, 9–50.
Janse, M., Kik, M.J.L., 2012. Mycobacterium avium granulomas in a captive epaulette shark, Hemiscyllium ocellatum (Bonnaterre). Journal of Fish Diseases 35, 935–940. https://doi.org/10.1111/j.1365-2761.2012.01444.x
Janse, M., Zimmerman, B., Geerlings, L., Brown, C., Nagelkerke, L.A.J., 2017. Sustainable species management of the elasmobranch populations within European aquariums: a conservation challenge. JZAR 5, 172–181. https://doi.org/10.19227/jzar.v5i4.313
Jensen, K., 2005. A monograph on the Lecanicephalidea (Platyhelminthes, Cestoda). Bulletin of the University of Nebraska State Museum, 18, 1–241.

Jensen, K., Caira, J.N., Cielocha, J.J., Littlewood, D.T.J., Waeschenbach, A., 2016. When proglottids and scoleces conflict: phylogenetic relationships and a family-level classification of the Lecanicephalidea (Platyhelminthes: Cestoda). International Journal for Parasitology 46, 291–310. https://doi.org/10.1016/j.ijpara.2016.02.002

Johnson, M.S., Kraver, D.W., Renshaw, G.M.C., Rummer, J.L., 2016. Will ocean acidification affect the early ontogeny of a tropical oviparous elasmobranch (Hemiscyllium ocellatum)? Conservation Physiology 4, cow003. https://doi.org/10.1093/conphys/cow003

Jørgensen, J.M., Pickles, J.O., 2002. The lateral line canal sensory organs of the Epaulette Shark (Hemiscyllium ocellatum). Acta Zoologica 83, 337–343. https://doi.org/10.1046/j.1463-6395.2002.00125.x

Kaneko, A., Lam, D.M.K., Wiesel, T.N., 1976. Isolated horizontal cells of elasmobranch retinae. Brain Research 105, 567–572. https://doi.org/10.1016/0006-8993(76)90605-3

Karsten, A. H., 2000. Stress effects on fecal corticosterone levels in the epaulette shark, Hemiscyllium ocellatum. Medical College of Ohio ProQuest Dissertations & Theses, 2000. 1402241.

Karsten, A.H., Turner JR., J.W., 2003. Fecal Corticosterone Assessment in the Epaulette Shark, Hemiscyllium ocellatum. Journal of Experimental Zoology Part A: Comparative Experimental Biology 299A, 188–196. https://doi.org/10.1002/jez.a.10300

Klimpfinger, C., Kriwet, J., 2020. Comparative morphology of labial cartilages in sharks (Chondrichthyes, Elasmobranchii). The European Zoological Journal 87, 741–753. https://doi.org/10.1080/24750263.2020.1844323

Klinard, N.V., Mull, C.G., Heithaus, M.R., MacNeil, M.A., 2025. Defining ecological roles of sharks on coral reefs. Biological Reviews 100, 2707–2725. https://doi.org/10.1111/brv.70065

Larson, H.K., Williams, R.S., Hammer, M.P., 2013. An annotated checklist of the fishes of the Northern Territory, Australia. Zootaxa 3696, 1–293. https://doi.org/10.11646/zootaxa.3696.1.1

Last, P.R., 2002. Freshwater and estuarine elasmobranchs of Australia. In Elasmobranch Biodiversity, Conservation and Management. Proceedings of the conference on Shark and Ray Biodiversity, Conservation and Management, Sabah, Malaysia, July 1997. IUCN, Gland, Switzerland: 185–193.

Last, P.R. & Stevens, J.D., 2009. Sharks and Rays of Australia. CSIRO Publishing, Collingwood, Vic.

Leigh, S.C., Summers, A.P., Hoffmann, S.L., German, D.P., 2021. Shark spiral intestines may operate as Tesla valves. Proceedings of the Royal Society B: Biological Sciences 288, 20211359. https://doi.org/10.1098/rspb.2021.1359

Leveelahti, L., Rytkönen, K.T., Renshaw, G.M.C., Nikinmaa, M., 2014. Revisiting redox-active antioxidant defenses in response to hypoxic challenge in both hypoxia-tolerant and hypoxia-sensitive fish species. Fish Physiology and Biochemistry 40, 183–191. https://doi.org/10.1007/s10695-013-9835-1

Lisney, T.J., Collin, S.P., 2007. Relative Eye Size in Elasmobranchs. Brain Behavior and Evolution 69, 266–279. https://doi.org/10.1159/000100036

Llitherland, L., Collin, S.P., 2008. Comparative visual function in elasmobranchs: Spatial arrangement and ecological correlates of photoreceptor and ganglion cell distributions. Visual Neuroscience 25, 549–561. https://doi.org/10.1017/S0952523808080693

Lonati, M., Jahanbakht, M., Atkins, D., Bierwagen, S.L., Chin, A., Barnett, A., Rummer, J.L., 2024. Novel use of deep neural networks on photographic identification of epaulette sharks (Hemiscyllium ocellatum) across life stages. Journal of Fish Biology 105, 1572–1587. https://doi.org/10.1111/jfb.15887

Marion, A.F.P., Condamine, F.L., Guinot, G., 2024. Sequential trait evolution did not drive deep-time diversification in sharks. Evolution 78, 1405–1425. https://doi.org/10.1093/evolut/qpae070

Martin, S.B., Downie, A.J., Cribb, T.H., 2020. A new subfamily for a clade of opecoelids (Trematoda: Digenea) exploiting marine fishes as second-intermediate hosts, with the first report of opecoelid metacercariae from an elasmobranch. Zoological Journal of the Linnean Society, 188(2), 455–472.

McAuley, R.B., Newbound, D.R., Ashworth, R., 2002. Field identification guide to Western Australian Sharks and Shark-like Rays. Fisheries Occasional Publications, 1: 1–25.

McKiernan, J.P., Grutter, A.S., Davies, A.J., 2005. Reproductive and feeding ecology of parasitic gnathiid isopods of epaulette sharks (Hemiscyllium ocellatum) with consideration of their role in the transmission of a haemogregarine. International Journal for Parasitology 35, 19–27. https://doi.org/10.1016/j.ijpara.2004.10.016

McMurrer, J., McElhiney, A., McNally, K., Innis, C.J., 2022. Observations on the use of passive integrated transponder (PIT) tags in teleosts and elasmobranchs at a public aquarium, 728 cases, 2007–2020. Zoo Biology 41, 576–581. https://doi.org/10.1002/zoo.21678

Mould, B. 1997. Classification of the recent Elasmobranchii. Copyright Brian Mould 1997.

Mull, C.G., Yopak, K.E., Dulvy, N.K., 2011. Does more maternal investment mean a larger brain? Evolutionary relationships between reproductive mode and brain size in chondrichthyans. Marine & Freshwater Research 62, 567–575. https://doi.org/10.1071/MF10145

Mulvey, J.M., Renshaw, G.M.C., 2009. GABA is not elevated during neuroprotective neuronal depression in the hypoxic epaulette shark (Hemiscyllium ocellatum), in: Comparative Biochemistry and Physiology Part A: Molecular & Integrative Physiology. pp. 273–277. https://doi.org/10.1016/j.cbpa.2008.10.017

Mulvey, J.M., Renshaw, G.M.C., 2000. Neuronal oxidative hypometabolism in the brainstem of the epaulette shark (*Hemiscyllium ocellatum)* in response to hypoxic pre-conditioning. Neuroscience Letters 290, 1–4. https://doi.org/10.1016/S0304-3940(00)01321-5

Nay, T.J., Longbottom, R.J., Gervais, C.R., Johansen, J.L., Steffensen, J.F., Rummer, J.L., Hoey, A.S., 2021. Regulate or tolerate: Thermal strategy of a coral reef flat resident, the epaulette shark, Hemiscyllium ocellatum. Journal of Fish Biology 98, 723–732. https://doi.org/10.1111/jfb.14616

Naylor, G.J.P., Caira, J.N., Jensen, K., Rosana, K.A.M., White, W.T., Last, P.R., 2012. A DNA sequence based approach to the identification of shark and ray species and its implications for global elasmobranch diversity and parasitology. Bulletin of the American Museum of Natural History, 367, 1–262.

Nevatte, R.J., Clark, J.A., Williamson, J.E., Gillings, M.R., 2019. The complete mitochondrial genome of the Epaulette Shark, Hemiscyllium ocellatum (Bonnaterre, 1788). Mitochondrial DNA Part B 4, 534–535. https://doi.org/10.1080/23802359.2018.1553511

Nilsson, G.E., Renshaw, G.M.C., 2004. Hypoxic survival strategies in two fishes: Extreme anoxia tolerance in the North European crucian carp and natural hypoxic preconditioning in a coral-reef shark. J EXP BIOL 207, 3131–3139. https://doi.org/10.1242/jeb.00979

Niwa, T., Uno, Y., Ohishi, Y., Kadota, M., Aburatani, N., Kiyatake, I., Katooka, D., Yorozu, M., Tsuzuki, N., Toyoda, A., Takagi, W., Nakamura, M., Kuraku, S., 2025. Sharks and rays have the oldest vertebrate sex chromosome with unique sex determination mechanisms. Proceedings of the National Academy of Sciences 122, e2513676122. https://doi.org/10.1073/pnas.2513676122

Osgood, G.J., Baum, J.K., 2015. Reef sharks: recent advances in ecological understanding to inform conservation. Journal of Fish Biology 87, 1489–1523. https://doi.org/10.1111/jfb.12839

Ota, Y., Erasmus, A., Grutter, A.S., Smit, N.J., 2024. Two new species and new host and distribution records of Gnathia Leach, 1814 (Crustacea, Isopoda, Gnathiidae) from Western Australia and the Great Barrier Reef, Australia. ZK 1193, 125–144. https://doi.org/10.3897/zookeys.1193.116538

Palm, H.W. 2004. The Trypanorhyncha Diesing, 1863. PKSPL–IPB Press ISBN 979–9336–39–2.

Payne, E.J., 2012. Husbandry and growth rates of neonate epaulette sharks, Hemiscyllium ocellatum in captivity. Zoo Biology 31, 718–724. https://doi.org/10.1002/zoo.20426

Peach, M. B., 2003. The behavioural role of pit organs in the epaulette shark. Journal of Fish Biology 62, 793–802. https://doi.org/10.1046/j.1095-8649.2003.00065.x

Peach, Meredith B., 2003. Inter- and intraspecific variation in the distribution and number of pit organs (free neuromasts) of sharks and rays. Journal of Morphology 256, 89–102. https://doi.org/10.1002/jmor.10078

Peach, M.B., 2002. Rheotaxis by epaulette sharks, Hemiscylliumocellatum (Chondrichthyes : Hemiscylliidae), on a coral reef flat. Australian Journal of Zoology 50, 407–414. https://doi.org/10.1071/ZO01081

Peach, M.B., Marshall, N.J., 2009. The comparative morphology of pit organs in elasmobranchs. Journal of Morphology 270, 688–701. https://doi.org/10.1002/jmor.10715

Peach, M.B., Marshall, N.J., 2000. The pit organs of elasmobranchs: a review. Philosophical Transactions of the Royal Society B: Biological Sciences 355, 1131–1134. https://doi.org/10.1098/rstb.2000.0653

Peach, M.B., Rouse, G.W., 2004. Phylogenetic trends in the abundance and distribution of pit organs of elasmobranchs. Acta Zoologica 85, 233–244. https://doi.org/10.1111/j.0001-7272.2004.00176.x

Porter, M.E., Hernandez, A.V., Gervais, C.R., Rummer, J.L., 2022. Aquatic Walking and Swimming Kinematics of Neonate and Juvenile Epaulette Sharks. Integrative and Comparative Biology 62, 1710–1724. https://doi.org/10.1093/icb/icac127

Porter, M.E., Roque, C.M., Long Jr., J.H., 2009. Turning maneuvers in sharks: Predicting body curvature from axial morphology. Journal of Morphology 270, 954–965. https://doi.org/10.1002/jmor.10732

Preston, E., Jönsson, A.-C., McManus, C.D., Conlon, J.M., Courtice, G.P., 1998. Comparative vascular responses in elasmobranchs to different structures of neuropeptide Y and peptide YY. Regulatory Peptides 78, 57–67. https://doi.org/10.1016/S0167-0115(98)00116-5

Preston, E., McManus, C.D., Jonsson, A.-C., Courtice, G.P., 1995. Vasoconstrictor effects of galanin and distribution of galanin containing fibres in three species of elasmobranch fish. Regulatory Peptides 58, 123–134. https://doi.org/10.1016/0167-0115(95)00060-O

Pridmore, P.A., 1995. Submerged walking in the epaulette shark Hemiscyllium ocellatum (Hemiscyllidae) and its implications for locomotion in rhipidistian fishes and early tetrapods. Zoology, 98, 278–297.

Raoult, V., Tosetto, L., Williamson, J.E., 2018. Drone-Based High-Resolution Tracking of Aquatic Vertebrates. Drones 2, 37. https://doi.org/10.3390/drones2040037

Raschi, W., Tabit, C., 1992. Functional aspects of Placoid Scales: A review and update. Australian Journal of Marine and Freshwater Research 43, 123–147. https://doi.org/10.1071/MF9920123

Renshaw, G.M.C., Neuzil, J., Girjes, A., 2004. Changes in gene expression in response to anoxic preconditioning. The Molecular Basis, 13.

Renshaw, G.M.C., Kerrisk, C.B., Nilsson, G.E., 2002. The role of adenosine in the anoxic survival of the epaulette shark, *Hemiscyllium ocellatum*. Comparative Biochemistry and Physiology Part B:

Biochemistry and Molecular Biology 131, 133–141. https://doi.org/10.1016/S1096-4959(01)00484-5
Renshaw, G.M.C., Wise, G., Dodd, P.R., 2010. Ecophysiology of neuronal metabolism in transiently oxygen-depleted environments: Evidence that GABA is accumulated pre-synaptically in the cerebellum. Comparative Biochemistry and Physiology Part A: Molecular & Integrative Physiology 155, 486–492. https://doi.org/10.1016/j.cbpa.2009.10.039
Riggs, C.L., Summers, A., Warren, D.E., Nilsson, G.E., Lefevre, S., Dowd, W.W., Milton, S., Podrabsky, J.E., 2018. Small Non-coding RNA Expression and Vertebrate Anoxia Tolerance. Frontiers in Genetics Volume 9-2018. https://doi.org/10.3389/fgene.2018.00230
Roff, G., Doropoulos, C., Rogers, A., Bozec, Y.-M., Krueck, N.C., Aurellado, E., Priest, M., Birrell, C., Mumby, P.J., 2016. The Ecological Role of Sharks on Coral Reefs. Trends in Ecology & Evolution 31, 395–407. https://doi.org/10.1016/j.tree.2016.02.014
Rosa, R., Rummer, J.L., Munday, P.L., 2017. Biological responses of sharks to ocean acidification. Biology Letters 13, 20160796. https://doi.org/10.1098/rsbl.2016.0796
Routley, M.H., Nilsson, G.E., Renshaw, G.M.C., 2002. Exposure to hypoxia primes the respiratory and metabolic responses of the epaulette shark to progressive hypoxia. Comparative Biochemistry and Physiology Part A: Molecular & Integrative Physiology 131, 313–321. https://doi.org/10.1016/S1095-6433(01)00484-6
Ryan, L.A., Chapuis, L., Hemmi, J.M., Collin, S.P., McCauley, R.D., Yopak, K.E., Gennari, E., Huveneers, C., Kempster, R.M., Kerr, C.C., Schmidt, C., Egeberg, C.A., Hart, N.S., 2017a. Effects of auditory and visual stimuli on shark feeding behaviour: the disco effect. Marine Biology 165, 11. https://doi.org/10.1007/s00227-017-3256-0
Ryan, L.A., Hemmi, J.M., Collin, S.P., Hart, N.S., 2017b. Electrophysiological measures of temporal resolution, contrast sensitivity and spatial resolving power in sharks. Journal of Comparative Physiology A 203, 197–210. https://doi.org/10.1007/s00359-017-1154-z
Rytkönen, K.T., Renshaw, G.M., Ashton, K.J., Williams-Pritchard, G., Leder, E.H., Nikinmaa, M., 2010. Elasmobranch qPCR reference genes: a case study of hypoxia preconditioned epaulette sharks. BMC Molecular Biology 11, 27. https://doi.org/10.1186/1471-2199-11-27
Rytkönen, K.T., Renshaw, G.M.C., Vainio, P.P., Ashton, K.J., Williams-Pritchard, G., Leder, E.H., Nikinmaa, M., 2012. Transcriptional responses to hypoxia are enhanced by recurrent hypoxia (hypoxic preconditioning) in the epaulette shark. PHYSIOL GENOMICS 44, 1090–1097. https://doi.org/10.1152/physiolgenomics.00081.2012
Sauer, D.J., Radford, C.A., Mull, C.G., Yopak, K.E., 2023. Quantitative assessment of inner ear variation in elasmobranchs. Scientific Reports 13, 11939. https://doi.org/10.1038/s41598-023-39151-0
Schieber, N.L., Collin, S.P., Hart, N.S., 2012. Comparative retinal anatomy in four species of elasmobranch. Journal of Morphology 273, 423–440. https://doi.org/10.1002/jmor.11033
Schluessel, V., Bennett, M.B., Bleckmann, H., Blomberg, S., Collin, S.P., 2008. Morphometric and ultrastructural comparison of the olfactory system in elasmobranchs: The significance of structure–function relationships based on phylogeny and ecology. Journal of Morphology 269, 1365–1386. https://doi.org/10.1002/jmor.10661
Schwieterman, G.D., Rummer, J.L., Bouyoucos, I.A., Bushnell, P.G., Brill, R.W., 2021. A lack of red blood cell swelling in five elasmobranch fishes following air exposure and exhaustive exercise. Comparative Biochemistry and Physiology Part A: Molecular & Integrative Physiology 258, 110978. https://doi.org/10.1016/j.cbpa.2021.110978

Sendell-Price, A.T., Tulenko, F.J., Pettersson, M., Kang, D., Montandon, M., Winkler, S., Kulb, K., Naylor, G.P., Phillippy, A., Fedrigo, O., Mountcastle, J., Balacco, J.R., Dutra, A., Dale, R.E., Haase, B., Jarvis, E.D., Myers, G., Burgess, S.M., Currie, P.D., Andersson, L., Schartl, M., 2023. Low mutation rate in epaulette sharks is consistent with a slow rate of evolution in sharks. Nat Commun 14, 6628. https://doi.org/10.1038/s41467-023-42238-x

Sherman, C.S., Simpfendorfer, C.A., Pacoureau, N., Matsushiba, J.H., Yan, H.F., Walls, R.H.L., Rigby, C.L., VanderWright, W.J., Jabado, R.W., Pollom, R.A., Carlson, J.K., Charvet, P., Bin Ali, A., Fahmi, Cheok, J., Derrick, D.H., Herman, K.B., Finucci, B., Eddy, T.D., Palomares, M.L.D., Avalos-Castillo, C.G., Kinattumkara, B., Blanco-Parra, M.-P., Dharmadi, Espinoza, M., Fernando, D., Haque, A.B., Mejía-Falla, P.A., Navia, A.F., Pérez-Jiménez, J.C., Utzurrum, J., Yuneni, R.R., Dulvy, N.K., 2023. Half a century of rising extinction risk of coral reef sharks and rays. Nature Communications 14, 15. https://doi.org/10.1038/s41467-022-35091-x

Simpfendorfer, C., A. Chin, A., Kyne, P., Rigby, C., Sherman, S., White, W. 2019. A Report Card for Australia's Sharks. https://www.sharkreportcard.org/.

Skov, P.V., Bennett, M.B., 2004. The secondary vascular system of Actinopterygii: interspecific variation in origins and investment. Zoomorphology 123, 55–64. https://doi.org/10.1007/s00435-003-0094-z

Soares, K.D.A., Mathubara, K., 2022. Combined phylogeny and new classification of catsharks (Chondrichthyes: Elasmobranchii: Carcharhiniformes). Zoological Journal of the Linnean Society 195, 761–814. https://doi.org/10.1093/zoolinnean/zlab108

Söderström, V., Renshaw, G.M.C., Nilsson, G.E., 1999. Brain blood flow and blood pressure during hypoxia in the epaulette shark Hemiscyllium ocellatum, a hypoxia-tolerant elasmobranch. Journal of Experimental Biology, 202(7), 829–835.

Speers-Roesch, B., Brauner, C.J., Farrell, A.P., Hickey, A.J.R., Renshaw, G.M.C., Wang, Y.S., Richards, J.G., 2012a. Hypoxia tolerance in elasmobranchs. II. Cardiovascular function and tissue metabolic responses during progressive and relative hypoxia exposures. Journal of Experimental Biology 215, 103–114. https://doi.org/10.1242/jeb.059667

Speers-Roesch, B., Richards, J.G., Brauner, C.J., Farrell, A.P., Hickey, A.J.R., Wang, Y.S., Renshaw, G.M.C., 2012b. Hypoxia tolerance in elasmobranchs. I. Critical oxygen tension as a measure of blood oxygen transport during hypoxia exposure. Journal of Experimental Biology 215, 93–102. https://doi.org/10.1242/jeb.059642

Stensløkken, K.-O., Sundin, L., Renshaw, G.M.C., Nilsson, G.E., 2004. Adenosinergic and cholinergic control mechanisms during hypoxia in the epaulette shark (Hemiscyllium ocellatum), with emphasis on branchial circulation. Journal of Experimental Biology 207, 4451–4461. https://doi.org/10.1242/jeb.01291

Sternes, P.C., Shimada, K., 2020. Body forms in sharks (Chondrichthyes: Elasmobranchii) and their functional, ecological, and evolutionary implications. Zoology 140, 125799. https://doi.org/10.1016/j.zool.2020.125799

Thomas, P.A., Peele, E.E., Wheeler, C.R., Yopak, K., Rummer, J.L., Mandelman, J.W., Kinsey, S.T., 2023. Effects of projected end-of-century temperature on the muscle development of neonate epaulette sharks, Hemiscyllium ocellatum. Mar Biol 170, 71. https://doi.org/10.1007/s00227-023-04218-z

Torralba Sáez, M., Hofreiter, M., Straube, N., 2024. Shark genome size evolution and its relationship with cellular, life-history, ecological, and diversity traits. Scientific Reports 14, 8909. https://doi.org/10.1038/s41598-024-59202-4

Travis, K. G., 2020. Comparative Biomechanics of Submerged and Partially Emerged Walking in the Epaulette Shark (Hemiscyllium ocellatum). California State University, Long Beach ProQuest Dissertations & Theses. 28001935.
VanderWright, W.J., Dudgeon, C.L., Erdmann, M.V., Sianipar, A., Dulvy, N.K., 2022. Extinction Risk and the Small Population Paradigm in the Micro-Endemic Radiation of Epaulette Sharks, in: DellaSala, D.A., Goldstein, M.I. (Eds.), Imperiled: The Encyclopedia of Conservation. Elsevier, Oxford, pp. 752–762. https://doi.org/10.1016/B978-0-12-821139-7.00130-6
Weigmann, S., 2016. Annotated checklist of the living sharks, batoids and chimaeras (Chondrichthyes) of the world, with a focus on biogeographical diversity. Journal of Fish Biology 88, 837–1037. https://doi.org/10.1111/jfb.12874
Wells, R.M.G., Baldwin, J., Ryder, J.M., 1992. Respiratory function and nucleotide composition of erythrocytes from tropical elasmobranchs. Comparative Biochemistry and Physiology Part A: Physiology 103, 157–162. https://doi.org/10.1016/0300-9629(92)90256-P
West, G.J. & Carter, S., 1990. Observations on the development and growth of the epaulette shark Hemiscyllium ocellatum (Bonnaterre) in captivity. Journal of Aquariculture and Aquatic Sciences, 4, 111–117.
Wheeler, A., 1986. Catalogue of the Natural History Drawings commissioned by Joseph Banks on the Endeavour Voyage 1768-1771 Held in the British Museum (Natural History). Bulletin of the British Museum of Natural History (Historical Series), 13, 1–172.
Wheeler, C.R., Awruch, C.A., Mandelman, J.W., Rummer, J.L., 2025. Assessing the metabolic and physiological costs of oviparity in the epaulette shark (Hemiscyllium ocellatum). Biology Open 14, bio062076. https://doi.org/10.1242/bio.062076
Wheeler, C.R., Irschick, D.J., Mandelman, J.W., Rummer, J.L., 2023. Nonlethally assessing elasmobranch ontogenetic shifts in energetics. Journal of Fish Biology 103, 235–246. https://doi.org/10.1111/jfb.15425
Wheeler, C.R., Lang, B.J., Mandelman, J.W., Rummer, J.L., 2022. The upper thermal limit of epaulette sharks (Hemiscyllium ocellatum) is conserved across three life history stages, sex and body size. Conservation Physiology 10, coac074. https://doi.org/10.1093/conphys/coac074
Wheeler, C.R., Rummer, J.L., 2024. Evidence of dystocia in an oviparous shark. Journal of Fish Biology 105, 1004–1007. https://doi.org/10.1111/jfb.15819
Wheeler, C.R., Rummer, J.L., Bailey, B., Lockwood, J., Vance, S., Mandelman, J.W., 2021. Future thermal regimes for epaulette sharks (Hemiscyllium ocellatum): growth and metabolic performance cease to be optimal. Sci Rep 11, 454. https://doi.org/10.1038/s41598-020-79953-0
Winther-Janson, M., Wueringer, B.E., Seymour, J.E., 2012. Electroreceptive and Mechanoreceptive Anatomical Specialisations in the Epaulette Shark (Hemiscyllium ocellatum). PLOS ONE 7, e49857. https://doi.org/10.1371/journal.pone.0049857
Wise, G., Dodd, P. R., Renshaw, G. M. C., 1998. The kinetics of specific tritiated flunitrazepam binding in the brain of the epaulette shark (Hemiscyllium ocellatum).
Wise, G., Mulvey, J.M., Renshaw, G.M.C., 1998. Hypoxia tolerance in the epaulette shark (Hemiscyllium ocellatum). J EXP ZOOL 281, 1–5. https://doi.org/10.1002/(SICI)1097-010X(19980501)281:1%253C1::AID-JEZ1%253E3.0.CO;2-S
Wosnick, N., Leite, R.D., Giareta, E.P., Morick, D., Musyl, M., 2022. Global assessment of shark strandings. Fish and Fisheries 23, 786–799. https://doi.org/10.1111/faf.12648
Wu, E.H., 1994. Kinematic analysis of jaw protrusion in orectolobiform sharks: A new mechanism for jaw protrusion in elasmobranchs. Journal of Morphology 222, 175–190. https://doi.org/10.1002/jmor.1052220205

Yopak, K.E., Lisney, T.J., 2012. Allometric Scaling of the Optic Tectum in Cartilaginous Fishes. Brain Behavior and Evolution 80, 108–126. https://doi.org/10.1159/000339875
Yopak, K.E., Lisney, T.J., Collin, S.P., 2015. Not all sharks are "swimming noses": variation in olfactory bulb size in cartilaginous fishes. Brain Structure and Function 220, 1127–1143. https://doi.org/10.1007/s00429-014-0705-0
Yopak, K.E., Lisney, T.J., Collin, S.P., Montgomery, J.C., 2007. Variation in Brain Organization and Cerebellar Foliation in Chondrichthyans: Sharks and Holocephalans. Brain Behavior and Evolution 69, 280–300. https://doi.org/10.1159/000100037
Yopak, K.E., Lisney, T.J., Darlington, R.B., Collin, S.P., Montgomery, J.C., Finlay, B.L., 2010. A conserved pattern of brain scaling from sharks to primates. Proceedings of the National Academy of Sciences 107, 12946–12951. https://doi.org/10.1073/pnas.1002195107

Supplemental table 1: sources used in Figure 1. Sources were compiled from publications presenting original or synthesized information on the *Hemiscyllium ocellatum*, identified primarily through Shark References and supplemented with Google Scholar search results. Publications that did not contribute original or synthesized data were excluded.

**Category**	**Reference**
Biology (general)	Allen, G.R., Erdmann, M.V., White, W.T., Fahmi, Dudgeon, C.L., 2016. Review of the bamboo shark genus Hemiscyllium (Orectolobiformes: Hemiscyllidae). Journal of the Ocean Science Foundation, 23, 51–97.
Conservation	Alves, L.M.F., Lemos, M.F.L., Cabral, H., Novais, S.C., 2022. Elasmobranchs as bioindicators of pollution in the marine environment. Marine Pollution Bulletin 176, 113418. https://doi.org/10.1016/j.marpolbul.2022.113418
Morphology	Appleby, M., Raoult, V., Broadhurst, M.K., Gaston, T., 2024. Can denticle morphology help identify southeastern Australian elasmobranchs? Journal of Fish Biology 104, 1848–1859. https://doi.org/10.1111/jfb.15704
Genomics	Ashton, K., Pritchard, G., Renshaw, G. M. C., Headrick, J. P., 2006. Fish and chips: Cardiac gene expression profiling in the anoxia tolerant epaulette shark, Journal of Molecular and Cellular Cardiology, 41733.
Morphology	Atkinson, C.J.L. & Collin, S.P., 2012. Structure and Topographic Distribution of Oral Denticles in Elasmobranch Fishes. Biological Bulletin, 222(1), 26–34.
Sensory biology	Atkinson, C.J.L., Collin, S.P., 2025. Surface morphology and distribution of oropharyngeal taste papillae in sharks and rays (Elasmobranchii, Chondrichthyes): Implications for gustatory sensitivity. Journal of Anatomy 247, 924–952. https://doi.org/10.1111/joa.14278
Ecology	Avrit, S., 2024. An Economic and Ecological Gap Analysis of Epaulette Sharks, Hemiscyllium. California State University, Long Beach.
Physiology	Baldwin, J., Wells, R.M.G., 1990. Oxygen transport potential in tropical elasmobranchs from the Great Barrier Reef: relationship between haematology and blood viscosity. Journal of Experimental Marine Biology and Ecology 144, 145–155. https://doi.org/10.1016/0022-0981(90)90025-8
Physiology	Becerril-García, E.E., Arellano-Martínez, M., Bernot-Simon, D., Hoyos-Padilla, E.M., Galván-Magaña, F., Godard-Codding, C., 2020. Steroid hormones and chondrichthyan reproduction: physiological functions, scientific research, and implications for conservation. PeerJ 8, e9686. https://doi.org/10.7717/peerj.9686
Parasitology	Bennett, M.B., Heupel, M.R., Bennett, S.M., Parker, A.R., 1997. Sheina orri (Myodocopa: Cypridinidae), an ostracod parasitic on the gills of the epaulette shark, Hemiscyllium ocellatum (Elasmobranchii: Hemiscyllidae). International Journal for Parasitology 27, 275–281. https://doi.org/10.1016/S0020-7519(96)00201-9
Physiology	Bouyoucos, I.A., 2021. Walking sharks cannot beat the heat. Conservation Physiology 9, coab035. https://doi.org/10.1093/conphys/coab035
Physiology	Bouyoucos, I.A., Simpfendorfer, C.A., Rummer, J.L., 2019. Estimating oxygen uptake rates to understand stress in sharks and rays. Rev Fish Biol Fisheries 29, 297–311. https://doi.org/10.1007/s11160-019-09553-3

Sensory biology	Bozzano, A., Collin, S.P., 2000. Retinal Ganglion Cell Topography in Elasmobranchs. Brain Behavior and Evolution 55, 191–208. https://doi.org/10.1159/000006652
Conservation	Buckley, K.A., Crook, D.A., Pillans, R.D., Smith, L., Kyne, P.M., 2018. Sustainability of threatened species displayed in public aquaria, with a case study of Australian sharks and rays. Reviews in Fish Biology and Fisheries 28, 137–151. https://doi.org/10.1007/s11160-017-9501-2
Parasitology	Burreson, E., 1989. Hematozoa of Fishes From Heron Island, Australia, With the Description of 2 New Species of Trypanosoma. Australian Journal of Zoology 37, 15–23. https://doi.org/10.1071/ZO9890015
Classification	Carpenter, K.E. & Niem, V.H., 1998. FAO species identification guide for fishery purposes. The living marine resources of the Western Central Pacific. Volume 2. Cephalopods, crustaceans, holothurians and sharks. Rome, FAO. 1998: 687–1396.
Conservation	Cavanagh, R.D., Kyne, P.M., Fowler, S.L., Musick, J.A., Bennett, M.B., 2003. The Conservation Status of Australasian Chondrichthyans. Report of the IUCN Shark Specialist Group Australia and Oceania Regional Red List Workshop. The University of Queensland, School of Biomedical Sciences, Brisbane, Australia.
Ecology	Ceccarelli, D.M., Frisch, A.J., Graham, N.A.J., Ayling, A.M., Beger, M., 2014. Habitat partitioning and vulnerability of sharks in the Great Barrier Reef Marine Park. Reviews in Fish Biology and Fisheries 24, 169–197. https://doi.org/10.1007/s11160-013-9324-8
Physiology	Chapman, C. A., 2009. Anoxia Survival Strategies in the Grey Carpet Shark (Chiloscyllium punctatum) and the Epaulette Shark (Hemiscyllium ocellatum). PhD Thesis, School of Physiotherapy and Exercise Science, Griffith University. https://doi.org/10.25904/1912/594.
Physiology	Chapman, C.A., Harahush, B.K., Renshaw, G.M.C., 2011. The physiological tolerance of the grey carpet shark (Chiloscyllium punctatum) and the epaulette shark (Hemiscyllium ocellatum) to anoxic exposure at three seasonal temperatures. Fish Physiol Biochem 37, 387–399. https://doi.org/10.1007/s10695-010-9439-y
Physiology	Chapman, C.A., Renshaw, G.M.C., 2009. Hematological responses of the grey carpet shark (Chiloscyllium punctatum) and the epaulette shark (Hemiscyllium ocellatum) to anoxia and re-oxygenation. Journal of Experimental Zoology Part A: Ecological Genetics and Physiology 311A, 422–438. https://doi.org/10.1002/jez.539
Classification	Compagno, L.J.V., 1984. FAO Species Catalogue. Vol 4: Sharks of the world, Part 1 - Hexanchiformes to Lamniformes. FAO Fisheries Synopsis No. 125, 4(1): 1–250.
Classification	Compagno, L.J.V., 2001. FAO Species Catalogue. Vol 2: Sharks of the world, Bullhead, mackerel and carpet sharks (Heterodontiformes, Lamniformes and Orectolobiformes). FAO Species Catalogue for Fishery Purposes No. 1, Vol. 2: 1–250.
Classification	Compagno, L.J.V., 2005. Checklist of living Chondrichthyes. In W.C. Hamlett (Ed.), Reproductive biology and phylogeny of chondrichthyes:

	sharks, rays and chimaeras, Vol. 3 (pp. 503–548). Science Publishers, Endfield, USA.
Classification	Compagno, L.J.V. & Niem, V.H., 2005. Part Sharks: Hexanchidae, Echinorhinidae, Squalidae, Squatinidae, Heterodontidae, Parascyllidae, Bracheluridae, Orectolobidae, Hemiscyllidae, Odontaspididae, Scyliorhinidae, Proscyllidae, Triakidae, In K.E. Carpenter and V.H. Niem(eds.), FAO Identification Guide for Fishery Purposes. The Living Marine Resources of the Western Central Pacific. Food and Agriculture Organization, Rome: 1195–1232, 1235–1259, 1264–1267, 1279–1295, 1297–1304, 1312–1360.
Morphology	da Silva, J.P.C.B., De Carvalho, M.R., 2015. Morphology and phylogenetic significance of the pectoral articular region in elasmobranchs (Chondrichthyes). Zoological Journal of the Linnean Society 175, 525–568. https://doi.org/10.1111/zoj.12287
Morphology	da Silva, J.P.C.B., Vaz, D.F.B., 2023. Morphology and phylogenetic significance of the pelvic articular region in elasmobranchs (Chondrichthyes). Cladistics 39, 155–197. https://doi.org/10.1111/cla.12528
Phylogeny	de Carvalho, M.R., 1996. Higher-Level Elasmobranch phylogeny, basal Squaleans, and paraphyly. In Stiassny, M.L.J., Parenti, L.R., & Johnson, G.D.(ed.), Interrelation of Fishes 3. Academic Press, New York. 35–62 pp.
Phylogeny	Devaux, J.B.L., Hickey, A.J.R., Renshaw, G.M.C., 2023. Succinate-mediated reactive oxygen species production in the anoxia-tolerant epaulette (Hemiscyllium ocellatum) and grey carpet (Chiloscyllium punctatum) sharks. Biology Letters 19, 20230344. https://doi.org/10.1098/rsbl.2023.0344
Phylogeny	Devaux, J.B.L., Hickey, A.J.R., Renshaw, G.M.C., 2019. Mitochondrial plasticity in the cerebellum of two anoxia-tolerant sharks: contrasting responses to anoxia/re-oxygenation. Journal of Experimental Biology 222, jeb191353. https://doi.org/10.1242/jeb.191353
Classification	Dingerkus, G. & de Fino, T.C., 1983. A revision of the orectolobiform shark family Hemiscyllidae (Chondrichthyes, Selachii). Bulletin of the American Museum of Natural History, 176(1), 1–93.
Physiology	Dowd, W.W., Renshaw, G.M.C., Cech, J.J., Kültz, D., 2010. Compensatory proteome adjustments imply tissue-specific structural and metabolic reorganization following episodic hypoxia or anoxia in the epaulette shark (Hemiscyllium ocellatum). Physiological Genomics 42, 93–114. https://doi.org/10.1152/physiolgenomics.00176.2009
Phylogeny	Dudgeon, C.L., Corrigan, S., Yang, L., Allen, G.R., Erdmann, M.V., Fahmi, Sugeha, H.Y., White, W.T., Naylor, G.J.P., 2020. Walking, swimming or hitching a ride? Phylogenetics and biogeography of the walking shark genus Hemiscyllium. Marine & Freshwater Research 71, 1107–1117. https://doi.org/10.1071/MF19163
Classification	Ebert, D.A, Fowler, S., Compagno, L.J.V., 2013. Sharks of the World – A fully illustrated guide. Wild Nature Press, ISBN 978–0–9573946–0–5: 528pp.
Parasitology	Eiras, J.C., Lu, Y.S., Gibson, D.I., Fiala, I., Saraiva, A., Cruz, C., Santos, M.J., 2012. Synopsis of the species of Chloromyxum Mingazzini, 1890

	(Myxozoa: Myxosporea: Chloromyxidae). Systematic Parasitology 83, 203–225. https://doi.org/10.1007/s11230-012-9380-9
Genomics	Ermakova, G.V., Meyntser, I.V., Zaraisky, A.G., Bayramov, A.V., 2024. Loss of noggin1, a classic embryonic inducer gene, in elasmobranchs. Scientific Reports 14, 3805. https://doi.org/10.1038/s41598-024-54435-9
Ecology	Fahmi, Putra, Y., Oktaviyani, S., Jutan, Y., Haris, A.S., Jamlaay, F., 2025. Ecology, conservation, and future perspective of walking sharks (Hemiscyllium spp.) in Indonesia. Pacific Conservation Biology 31, PC24088. https://doi.org/10.1071/PC24088
Physiology	Fuery, C.J., Attwood, P.V., Withers, P.C., Yancey, P.H., Baldwin, J., Guppy, M., 1997. Effects of Urea on M4-Lactate Dehydrogenase from Elasmobranchs and Urea-Accumulating Australian Desert Frogs. Comparative Biochemistry and Physiology Part B: Biochemistry and Molecular Biology 117, 143–150. https://doi.org/10.1016/S0305-0491(96)00287-8
Sensory biology	Gauthier, A.R.G., Whitehead, D.L., Tibbetts, I.R., Bennett, M.B., 2019. Comparative morphology of the electrosensory system of the epaulette shark Hemiscyllium ocellatum and brown-banded bamboo shark Chiloscyllium punctatum. Journal of Fish Biology 94, 313–319. https://doi.org/10.1111/jfb.13893
Evolutionary biology	Gayford, J.H., Brazeau, M.D., Naylor, G.J.P., 2024. Evolutionary trends in the elasmobranch neurocranium. Scientific Reports 14, 11471. https://doi.org/10.1038/s41598-024-62004-3
Physiology	Gayford, J.H., Rummer, J.L., 2025. Tonic immobility in cartilaginous fishes (Chondrichthyes): function, evolutionary history, and future directions. Reviews in Fish Biology and Fisheries 35, 1301–1315. https://doi.org/10.1007/s11160-025-09958-3
Developmenta l biology	Gervais, C., Mourier, J., Rummer, J.L., 2016. Developing in warm water: irregular colouration and patterns of a neonate elasmobranch. Marine Biodiversity 46, 743–744. https://doi.org/10.1007/s12526-015-0429-2
Physiology	Gervais, C.R., Nay, T.J., Renshaw, G., Johansen, J.L., Steffensen, J.F., Rummer, J.L., 2018. Too hot to handle? Using movement to alleviate effects of elevated temperatures in a benthic elasmobranch, Hemiscyllium ocellatum. Mar Biol 165, 162. https://doi.org/10.1007/s00227-018-3427-7
Parasitology	Gleeson, R.J., Adlard, R.D., 2012. Phylogenetic relationships amongst Chloromyxum Mingazzini, 1890 (Myxozoa: Myxosporea), and the description of six novel species from Australian elasmobranchs. Parasitology International 61, 267–274. https://doi.org/10.1016/j.parint.2011.10.008
Parasitology	Gleeson, R.J., Bennet, M.B., Adlard, R.D., 2010. First taxonomic description of multivalvulidan myxosporean parasites from elasmobranchs: Kudoa hemiscylli n.sp. and Kudoa carcharhini n.sp. (Myxosporea: Multivalvulidae). Parasitology 137, 1885–1898. https://doi.org/10.1017/S0031182010000855
Morphology	Goto, T., Nishida, K., Nakaya, K., 1999. Internal morphology and function of paired fins in the epaulette shark,hemiscyllium ocellatum. Ichthyological Research 46, 281–287. https://doi.org/10.1007/BF02678514

Classification	Goto, T., 2001. Comparative Anatomy, Phylogeny and Cladistic Classification of the order Orectolobiformes (Chondrichthyes, Elasmobranchii). Memoirs of the Graduate School of Fisheries Sciences, Hokkaido University, 48, 1–100.
Sensory biology	Grew, M., Raoult, V., Gaston, T.F., 2024. Behavioural response of benthic elasmobranchs to a neodymium magnet under controlled laboratory conditions. Fisheries Research 271, 106926. https://doi.org/10.1016/j.fishres.2023.106926
Evolutionary biology	Guinot, G., Cavin, L., 2020. Distinct Responses of Elasmobranchs and Ray-Finned Fishes to Long-Term Global Change. Frontiers in Ecology and Evolution Volume 7-2019. https://doi.org/10.3389/fevo.2019.00513
Genomics	Hardie, D.C., Hebert, P.D.N., 2003. The nucleotypic effects of cellular DNA content in cartilaginous and ray-finned fishes. Genome 46, 683–706. https://doi.org/10.1139/g03-040
Sensory biology	Hart, N.S., Theiss, S.M., Harahush, B.K., Collin, S.P., 2011. Microspectrophotometric evidence for cone monochromacy in sharks. Naturwissenschaften 98, 193–201. https://doi.org/10.1007/s00114-010-0758-8
Veterinary science	Hasenei, A., Foyle, L., Rummer, J., 2025. A novel, nonlethal liver biopsy procedure in an elasmobranch. Australian Veterinary Journal 103, 407–410. https://doi.org/10.1111/avj.13432
Physiology	Heinrich, D.D.U., Rummer, J.L., Morash, A.J., Watson, S.-A., Simpfendorfer, C.A., Heupel, M.R., Munday, P.L., 2014. A product of its environment: the epaulette shark (Hemiscyllium ocellatum) exhibits physiological tolerance to elevated environmental CO(2). Conservation Physiology 2, cou047. https://doi.org/10.1093/conphys/cou047
Physiology	Heinrich, D.D.U., Watson, S.-A., Rummer, J.L., Brandl, S.J., Simpfendorfer, C.A., Heupel, M.R., Munday, P.L., 2016. Foraging behaviour of the epaulette shark Hemiscyllium ocellatum is not affected by elevated CO2. ICES Journal of Marine Science 73, 633–640. https://doi.org/10.1093/icesjms/fsv085
Morphology	Herman, J., Hovestadt-Euler, M., Hovestadt, D.C., 1992. Contributions to the study of the comparative morphology of teeth and other relevant ichthyodorulites in living superspecific taxa of Chondrichthyan fishes. Part A: Selachii. No. 4: Order: Orectolobiformes Families: Brachaeluridae, Giglymostomatidae, Hemiscylliidae, Orectolobidae, Parascylliidae, Rhiniodontidae, Stegostomatidae. Order: Pristiophoriformes - Family: Pristiophoridae Order: Squatiniformes - Family: Squatinidae. Bulletin de l'Institut Royal des Sciences Naturelles de Belgique, Biologie, 62, 193–254.
Life history	Heupel, M. R., 1999. Life history of the epaulette shark Hemiscyllium ocellatum on Heron Island Reef, Great Barrier Reef, Australia, with comments on other reef sharks. PhD Thesis, School of Biomedical Sciences, The University of Queensland. https://doi.org/10.14264/734685.
Ecology	Heupel, M.R., Bennett, M.B., 2007. Estimating Abundance of Reef-Dwelling Sharks: A Case Study of the Epaulette Shark, Hemiscyllium ocellatum (Elasmobranchii: Hemiscyllidae)1. pasc 61, 383–394.

	https://doi.org/10.2984/1534-6188(2007)61%255B383:EAORSA%255D2.0.CO;2
Parasitology	Heupel, M.R., Bennett, M.B., 1999. The occurrence, distribution and pathology associated with gnathiid isopod larvae infecting the epaulette shark, Hemiscyllium ocellatum. International Journal for Parasitology 29, 321–330. https://doi.org/10.1016/S0020-7519(98)00218-5
Behavioural ecology	Heupel, M.R., Bennett, M.B., 1998a. Observations on the diet and feeding habits of the epaulette shark, Hemiscyllium ocellatum (Bonnaterre), on Heron Island Reef, Great Barrier Reef, Australia. Mar. Freshwater Res. 49, 753–756. https://doi.org/10.1071/mf97026
Parasitology	Heupel, M.R., Bennett, M.B., 1998b. Infection of the epaulette shark, Hemiscyllium ocellatum (Bonnaterre), by the nematode parasite Proleptus australis Bayliss (Spirurida: Physalopteridae). Journal of Fish Diseases 21, 407–414. https://doi.org/10.1046/j.1365-2761.1998.00121.x
Histology	Heupel, M.R., Bennett, M.B., 1997. Histology of dart tag insertion sites in the epaulette shark. Journal of Fish Biology 50, 1034–1041. https://doi.org/10.1111/j.1095-8649.1997.tb01628.x
Parasitology	Heupel, M.R., Bennett, M.B., 1996. A myxosporean parasite (Myxosporea: Multivalvulida) in the skeletal muscle of epaulette sharks, Hemiscyllium ocellatum (Bonnaterre), from the Great Barrier Reef. Journal of Fish Diseases 19, 189–191. https://doi.org/10.1111/j.1365-2761.1996.tb00700.x
Morphology	Heupel, M. R., Simpfendorfer, C.A., Bennett, M.B., 1999. Skeletal deformities in elasmobranchs from Australian waters. Journal of Fish Biology 54, 1111–1115. https://doi.org/10.1111/j.1095-8649.1999.tb00861.x
Physiology	Heupel, M.R., Whittier, J.M., Bennett, M.B., 1999. Plasma steroid hormone profiles and reproductive biology of the epaulette shark, Hemiscyllium ocellatum. Journal of Experimental Zoology 284, 586–594. https://doi.org/10.1002/(SICI)1097-010X(19991001)284:5%253C586::AID-JEZ14%253E3.0.CO;2-B
Physiology	Hickey, A.J.R., Renshaw, G.M.C., Speers-Roesch, B., Richards, J.G., Wang, Y., Farrell, A.P., Brauner, C.J., 2012. A radical approach to beating hypoxia: depressed free radical release from heart fibres of the hypoxia-tolerant epaulette shark (Hemiscyllum ocellatum). J Comp Physiol B 182, 91–100. https://doi.org/10.1007/s00360-011-0599-6
Classification	Hutchins, J.B., 2001. Checklist of the fishes of Western Australia. Records of the Western Australian Museum, Supplement 63, 9–50.
Parasitology	Janse, M., Kik, M.J.L., 2012. Mycobacterium avium granulomas in a captive epaulette shark, Hemiscyllium ocellatum (Bonnaterre). Journal of Fish Diseases 35, 935–940. https://doi.org/10.1111/j.1365-2761.2012.01444.x
Conservation	Janse, M., Zimmerman, B., Geerlings, L., Brown, C., Nagelkerke, L.A.J., 2017. Sustainable species management of the elasmobranch populations within European aquariums: a conservation challenge. JZAR 5, 172–181. https://doi.org/10.19227/jzar.v5i4.313
Parasitology	Jensen, K., 2005. A monograph on the Lecanicephalidea (Platyhelminthes, Cestoda). Bulletin of the University of Nebraska State Museum, 18, 1–241.

Parasitology	Jensen, K., Caira, J.N., Cielocha, J.J., Littlewood, D.T.J., Waeschenbach, A., 2016. When proglottids and scoleces conflict: phylogenetic relationships and a family-level classification of the Lecanicephalidea (Platyhelminthes: Cestoda). International Journal for Parasitology 46, 291–310. https://doi.org/10.1016/j.ijpara.2016.02.002
Developmenta l biology	Johnson, M.S., Kraver, D.W., Renshaw, G.M.C., Rummer, J.L., 2016. Will ocean acidification affect the early ontogeny of a tropical oviparous elasmobranch (Hemiscyllium ocellatum)? Conservation Physiology 4, cow003. https://doi.org/10.1093/conphys/cow003
Sensory biology	Jørgensen, J.M., Pickles, J.O., 2002. The lateral line canal sensory organs of the Epaulette Shark (Hemiscyllium ocellatum). Acta Zoologica 83, 337–343. https://doi.org/10.1046/j.1463-6395.2002.00125.x
Sensory biology	Kaneko, A., Lam, D.M.K., Wiesel, T.N., 1976. Isolated horizontal cells of elasmobranch retinae. Brain Research 105, 567–572. https://doi.org/10.1016/0006-8993(76)90605-3
Physiology	Karsten, A. H., 2000. Stress effects on fecal corticosterone levels in the epaulette shark, Hemiscyllium ocellatum. Medical College of Ohio ProQuest Dissertations & Theses, 2000. 1402241.
Physiology	Karsten, A.H., Turner JR., J.W., 2003. Fecal Corticosterone Assessment in the Epaulette Shark, Hemiscyllium ocellatum. Journal of Experimental Zoology Part A: Comparative Experimental Biology 299A, 188–196. https://doi.org/10.1002/jez.a.10300
Morphology	Klimpfinger, C., Kriwet, J., 2020. Comparative morphology of labial cartilages in sharks (Chondrichthyes, Elasmobranchii). The European Zoological Journal 87, 741–753. https://doi.org/10.1080/24750263.2020.1844323
Ecology	Klinard, N.V., Mull, C.G., Heithaus, M.R., MacNeil, M.A., 2025. Defining ecological roles of sharks on coral reefs. Biological Reviews 100, 2707–2725. https://doi.org/10.1111/brv.70065
Classification	Larson, H.K., Williams, R.S., Hammer, M.P., 2013. An annotated checklist of the fishes of the Northern Territory, Australia. Zootaxa 3696, 1–293. https://doi.org/10.11646/zootaxa.3696.1.1
Classification	Last, P.R., 2002. Freshwater and estuarine elasmobranchs of Australia. In Elasmobranch Biodiversity, Conservation and Management. Proceedings of the conference on Shark and Ray Biodiversity, Conservation and Management, Sabah, Malaysia, July 1997. IUCN, Gland, Switzerland: 185–193.
Classification	Last, P.R. & Stevens, J.D., 2009. Sharks and Rays of Australia. CSIRO Publishing, Collingwood, Vic.
Technology	Leigh, S.C., Summers, A.P., Hoffmann, S.L., German, D.P., 2021. Shark spiral intestines may operate as Tesla valves. Proceedings of the Royal Society B: Biological Sciences 288, 20211359. https://doi.org/10.1098/rspb.2021.1359
Physiology	Leveelahti, L., Rytkönen, K.T., Renshaw, G.M.C., Nikinmaa, M., 2014. Revisiting redox-active antioxidant defenses in response to hypoxic challenge in both hypoxia-tolerant and hypoxia-sensitive fish species. Fish

	Physiology and Biochemistry 40, 183–191. https://doi.org/10.1007/s10695-013-9835-1
Morphology	Lisney, T.J., Collin, S.P., 2007. Relative Eye Size in Elasmobranchs. Brain Behavior and Evolution 69, 266–279. https://doi.org/10.1159/000100036
Sensory biology	Llitherland, L., Collin, S.P., 2008. Comparative visual function in elasmobranchs: Spatial arrangement and ecological correlates of photoreceptor and ganglion cell distributions. Visual Neuroscience 25, 549–561. https://doi.org/10.1017/S0952523808080693
Technology	Lonati, M., Jahanbakht, M., Atkins, D., Bierwagen, S.L., Chin, A., Barnett, A., Rummer, J.L., 2024. Novel use of deep neural networks on photographic identification of epaulette sharks (Hemiscyllium ocellatum) across life stages. Journal of Fish Biology 105, 1572–1587. https://doi.org/10.1111/jfb.15887
Genomics	Marion, A.F.P., Condamine, F.L., Guinot, G., 2024. Sequential trait evolution did not drive deep-time diversification in sharks. Evolution 78, 1405–1425. https://doi.org/10.1093/evolut/qpae070
Parasitology	Martin, S.B., Downie, A.J., Cribb, T.H., 2020. A new subfamily for a clade of opecoelids (Trematoda: Digenea) exploiting marine fishes as second-intermediate hosts, with the first report of opecoelid metacercariae from an elasmobranch. Zoological Journal of the Linnean Society, 188(2), 455–472.
Classification	McAuley, R.B., Newbound, D.R., Ashworth, R., 2002. Field identification guide to Western Australian Sharks and Shark-like Rays. Fisheries Occasional Publications, 1: 1–25.
Parasitology	McKiernan, J.P., Grutter, A.S., Davies, A.J., 2005. Reproductive and feeding ecology of parasitic gnathiid isopods of epaulette sharks (Hemiscyllium ocellatum) with consideration of their role in the transmission of a haemogregarine. International Journal for Parasitology 35, 19–27. https://doi.org/10.1016/j.ijpara.2004.10.016
Conservation	McMurrer, J., McElhiney, A., McNally, K., Innis, C.J., 2022. Observations on the use of passive integrated transponder (PIT) tags in teleosts and elasmobranchs at a public aquarium, 728 cases, 2007–2020. Zoo Biology 41, 576–581. https://doi.org/10.1002/zoo.21678
Classification	Mould, B. 1997. Classification of the recent Elasmobranchii. Copyright Brian Mould 1997.
Comparative anatomy	Mull, C.G., Yopak, K.E., Dulvy, N.K., 2011. Does more maternal investment mean a larger brain? Evolutionary relationships between reproductive mode and brain size in chondrichthyans. Marine & Freshwater Research 62, 567–575. https://doi.org/10.1071/MF10145
Physiology	Mulvey, J.M., Renshaw, G.M.C., 2009. GABA is not elevated during neuroprotective neuronal depression in the hypoxic epaulette shark (Hemiscyllium ocellatum), in: Comparative Biochemistry and Physiology Part A: Molecular & Integrative Physiology. pp. 273–277. https://doi.org/10.1016/j.cbpa.2008.10.017
Physiology	Mulvey, J.M., Renshaw, G.M.C., 2000. Neuronal oxidative hypometabolism in the brainstem of the epaulette shark (Hemiscyllium ocellatum) in response

	to hypoxic pre-conditioning. Neuroscience Letters 290, 1–4. https://doi.org/10.1016/S0304-3940(00)01321-5
Physiology	Nay, T.J., Longbottom, R.J., Gervais, C.R., Johansen, J.L., Steffensen, J.F., Rummer, J.L., Hoey, A.S., 2021. Regulate or tolerate: Thermal strategy of a coral reef flat resident, the epaulette shark, Hemiscyllium ocellatum. Journal of Fish Biology 98, 723–732. https://doi.org/10.1111/jfb.14616
Genomics	Naylor, G.J.P., Caira, J.N., Jensen, K., Rosana, K.A.M., White, W.T., Last, P.R., 2012. A DNA sequence based approach to the identification of shark and ray species and its implications for global elasmobranch diversity and parasitology. Bulletin of the American Museum of Natural History, 367, 1–262.
Genomics	Nevatte, R.J., Clark, J.A., Williamson, J.E., Gillings, M.R., 2019. The complete mitochondrial genome of the Epaulette Shark, Hemiscyllium ocellatum (Bonnaterre, 1788). Mitochondrial DNA Part B 4, 534–535. https://doi.org/10.1080/23802359.2018.1553511
Physiology	Nilsson, G.E., Renshaw, G.M.C., 2004. Hypoxic survival strategies in two fishes: Extreme anoxia tolerance in the North European crucian carp and natural hypoxic preconditioning in a coral-reef shark. J EXP BIOL 207, 3131–3139. https://doi.org/10.1242/jeb.00979
Genomics	Niwa, T., Uno, Y., Ohishi, Y., Kadota, M., Aburatani, N., Kiyatake, I., Katooka, D., Yorozu, M., Tsuzuki, N., Toyoda, A., Takagi, W., Nakamura, M., Kuraku, S., 2025. Sharks and rays have the oldest vertebrate sex chromosome with unique sex determination mechanisms. Proceedings of the National Academy of Sciences 122, e2513676122. https://doi.org/10.1073/pnas.2513676122
Ecology	Osgood, G.J., Baum, J.K., 2015. Reef sharks: recent advances in ecological understanding to inform conservation. Journal of Fish Biology 87, 1489–1523. https://doi.org/10.1111/jfb.12839
Parasitology	Ota, Y., Erasmus, A., Grutter, A.S., Smit, N.J., 2024. Two new species and new host and distribution records of Gnathia Leach, 1814 (Crustacea, Isopoda, Gnathiidae) from Western Australia and the Great Barrier Reef, Australia. ZK 1193, 125–144. https://doi.org/10.3897/zookeys.1193.116538
Parasitology	Palm, H.W. 2004. The Trypanorhyncha Diesing, 1863. PKSPL–IPB Press ISBN 979–9336–39–2.
Developmental biology	Payne, E.J., 2012. Husbandry and growth rates of neonate epaulette sharks, Hemiscyllium ocellatum in captivity. Zoo Biology 31, 718–724. https://doi.org/10.1002/zoo.20426
Sensory biology	Peach, M. B., 2003. The behavioural role of pit organs in the epaulette shark. Journal of Fish Biology 62, 793–802. https://doi.org/10.1046/j.1095-8649.2003.00065.x
Sensory biology	Peach, Meredith B., 2003. Inter- and intraspecific variation in the distribution and number of pit organs (free neuromasts) of sharks and rays. Journal of Morphology 256, 89–102. https://doi.org/10.1002/jmor.10078
Behavioural ecology	Peach, M.B., 2002. Rheotaxis by epaulette sharks, Hemiscylliumocellatum (Chondrichthyes : Hemiscylliidae), on a coral reef flat. Australian Journal of Zoology 50, 407–414. https://doi.org/10.1071/ZO01081

Sensory biology	Peach, M.B., Marshall, N.J., 2009. The comparative morphology of pit organs in elasmobranchs. Journal of Morphology 270, 688–701. https://doi.org/10.1002/jmor.10715
Sensory biology	Peach, M.B., Marshall, N.J., 2000. The pit organs of elasmobranchs: a review. Philosophical Transactions of the Royal Society B: Biological Sciences 355, 1131–1134. https://doi.org/10.1098/rstb.2000.0653
Sensory biology	Peach, M.B., Rouse, G.W., 2004. Phylogenetic trends in the abundance and distribution of pit organs of elasmobranchs. Acta Zoologica 85, 233–244. https://doi.org/10.1111/j.0001-7272.2004.00176.x
Kinematics	Porter, M.E., Hernandez, A.V., Gervais, C.R., Rummer, J.L., 2022. Aquatic Walking and Swimming Kinematics of Neonate and Juvenile Epaulette Sharks. Integrative and Comparative Biology 62, 1710–1724. https://doi.org/10.1093/icb/icac127
Kinematics	Porter, M.E., Roque, C.M., Long Jr., J.H., 2009. Turning maneuvers in sharks: Predicting body curvature from axial morphology. Journal of Morphology 270, 954–965. https://doi.org/10.1002/jmor.10732
Physiology	Preston, E., Jönsson, A.-C., McManus, C.D., Conlon, J.M., Courtice, G.P., 1998. Comparative vascular responses in elasmobranchs to different structures of neuropeptide Y and peptide YY. Regulatory Peptides 78, 57–67. https://doi.org/10.1016/S0167-0115(98)00116-5
Physiology	Preston, E., McManus, C.D., Jonsson, A.-C., Courtice, G.P., 1995. Vasoconstrictor effects of galanin and distribution of galanin containing fibres in three species of elasmobranch fish. Regulatory Peptides 58, 123–134. https://doi.org/10.1016/0167-0115(95)00060-O
Kinematics	Pridmore, P.A., 1995. Submerged walking in the epaulette shark Hemiscyllium ocellatum (Hemiscyllidae) and its implications for locomotion in rhipidistian fishes and early tetrapods. Zoology, 98, 278–297.
Ecology	Raoult, V., Tosetto, L., Williamson, J.E., 2018. Drone-Based High-Resolution Tracking of Aquatic Vertebrates. Drones 2, 37. https://doi.org/10.3390/drones2040037
Morphology	Raschi, W., Tabit, C., 1992. Functional aspects of Placoid Scales: A review and update. Australian Journal of Marine and Freshwater Research 43, 123–147. https://doi.org/10.1071/MF9920123
Physiology	Renshaw, G.M.C., Neuzil, J., Girjes, A., 2004. Changes in gene expression in response to anoxic preconditioning. The Molecular Basis, 13.
Physiology	Renshaw, G.M.C., Kerrisk, C.B., Nilsson, G.E., 2002. The role of adenosine in the anoxic survival of the epaulette shark, Hemiscyllium ocellatum. Comparative Biochemistry and Physiology Part B: Biochemistry and Molecular Biology 131, 133–141. https://doi.org/10.1016/S1096-4959(01)00484-5
Physiology	Renshaw, G.M.C., Wise, G., Dodd, P.R., 2010. Ecophysiology of neuronal metabolism in transiently oxygen-depleted environments: Evidence that GABA is accumulated pre-synaptically in the cerebellum. Comparative Biochemistry and Physiology Part A: Molecular & Integrative Physiology 155, 486–492. https://doi.org/10.1016/j.cbpa.2009.10.039

Genomics	Riggs, C.L., Summers, A., Warren, D.E., Nilsson, G.E., Lefevre, S., Dowd, W.W., Milton, S., Podrabsky, J.E., 2018. Small Non-coding RNA Expression and Vertebrate Anoxia Tolerance. Frontiers in Genetics Volume 9-2018. https://doi.org/10.3389/fgene.2018.00230
Ecology	Roff, G., Doropoulos, C., Rogers, A., Bozec, Y.-M., Krueck, N.C., Aurellado, E., Priest, M., Birrell, C., Mumby, P.J., 2016. The Ecological Role of Sharks on Coral Reefs. Trends in Ecology & Evolution 31, 395–407. https://doi.org/10.1016/j.tree.2016.02.014
Physiology	Rosa, R., Rummer, J.L., Munday, P.L., 2017. Biological responses of sharks to ocean acidification. Biology Letters 13, 20160796. https://doi.org/10.1098/rsbl.2016.0796
Physiology	Routley, M.H., Nilsson, G.E., Renshaw, G.M.C., 2002. Exposure to hypoxia primes the respiratory and metabolic responses of the epaulette shark to progressive hypoxia. Comparative Biochemistry and Physiology Part A: Molecular & Integrative Physiology 131, 313–321. https://doi.org/10.1016/S1095-6433(01)00484-6
Sensory biology	Ryan, L.A., Chapuis, L., Hemmi, J.M., Collin, S.P., McCauley, R.D., Yopak, K.E., Gennari, E., Huveneers, C., Kempster, R.M., Kerr, C.C., Schmidt, C., Egeberg, C.A., Hart, N.S., 2017a. Effects of auditory and visual stimuli on shark feeding behaviour: the disco effect. Marine Biology 165, 11. https://doi.org/10.1007/s00227-017-3256-0
Physiology	Ryan, L.A., Hemmi, J.M., Collin, S.P., Hart, N.S., 2017b. Electrophysiological measures of temporal resolution, contrast sensitivity and spatial resolving power in sharks. Journal of Comparative Physiology A 203, 197–210. https://doi.org/10.1007/s00359-017-1154-z
Genomics	Rytkönen, K.T., Renshaw, G.M., Ashton, K.J., Williams-Pritchard, G., Leder, E.H., Nikinmaa, M., 2010. Elasmobranch qPCR reference genes: a case study of hypoxia preconditioned epaulette sharks. BMC Molecular Biology 11, 27. https://doi.org/10.1186/1471-2199-11-27
Genomics	Rytkönen, K.T., Renshaw, G.M.C., Vainio, P.P., Ashton, K.J., Williams-Pritchard, G., Leder, E.H., Nikinmaa, M., 2012. Transcriptional responses to hypoxia are enhanced by recurrent hypoxia (hypoxic preconditioning) in the epaulette shark. PHYSIOL GENOMICS 44, 1090–1097. https://doi.org/10.1152/physiolgenomics.00081.2012
Sensory biology	Sauer, D.J., Radford, C.A., Mull, C.G., Yopak, K.E., 2023. Quantitative assessment of inner ear variation in elasmobranchs. Scientific Reports 13, 11939. https://doi.org/10.1038/s41598-023-39151-0
Comparative anatomy	Schieber, N.L., Collin, S.P., Hart, N.S., 2012. Comparative retinal anatomy in four species of elasmobranch. Journal of Morphology 273, 423–440. https://doi.org/10.1002/jmor.11033
Sensory biology	Schluessel, V., Bennett, M.B., Bleckmann, H., Blomberg, S., Collin, S.P., 2008. Morphometric and ultrastructural comparison of the olfactory system in elasmobranchs: The significance of structure–function relationships based on phylogeny and ecology. Journal of Morphology 269, 1365–1386. https://doi.org/10.1002/jmor.10661

Physiology	Schwieterman, G.D., Rummer, J.L., Bouyoucos, I.A., Bushnell, P.G., Brill, R.W., 2021. A lack of red blood cell swelling in five elasmobranch fishes following air exposure and exhaustive exercise. Comparative Biochemistry and Physiology Part A: Molecular & Integrative Physiology 258, 110978. https://doi.org/10.1016/j.cbpa.2021.110978
Genomics	Sendell-Price, A.T., Tulenko, F.J., Pettersson, M., Kang, D., Montandon, M., Winkler, S., Kulb, K., Naylor, G.P., Phillippy, A., Fedrigo, O., Mountcastle, J., Balacco, J.R., Dutra, A., Dale, R.E., Haase, B., Jarvis, E.D., Myers, G., Burgess, S.M., Currie, P.D., Andersson, L., Schartl, M., 2023. Low mutation rate in epaulette sharks is consistent with a slow rate of evolution in sharks. Nat Commun 14, 6628. https://doi.org/10.1038/s41467-023-42238-x
Conservation	Sherman, C.S., Simpfendorfer, C.A., Pacoureau, N., Matsushiba, J.H., Yan, H.F., Walls, R.H.L., Rigby, C.L., VanderWright, W.J., Jabado, R.W., Pollom, R.A., Carlson, J.K., Charvet, P., Bin Ali, A., Fahmi, Cheok, J., Derrick, D.H., Herman, K.B., Finucci, B., Eddy, T.D., Palomares, M.L.D., Avalos-Castillo, C.G., Kinattumkara, B., Blanco-Parra, M.-P., Dharmadi, Espinoza, M., Fernando, D., Haque, A.B., Mejía-Falla, P.A., Navia, A.F., Pérez-Jiménez, J.C., Utzurrum, J., Yuneni, R.R., Dulvy, N.K., 2023. Half a century of rising extinction risk of coral reef sharks and rays. Nature Communications 14, 15. https://doi.org/10.1038/s41467-022-35091-x
Conservation	Simpfendorfer, C., A. Chin, A., Kyne, P., Rigby, C., Sherman, S., White, W. 2019. A Report Card for Australia's Sharks. https://www.sharkreportcard.org/.
Comparative anatomy	Skov, P.V., Bennett, M.B., 2004. The secondary vascular system of Actinopterygii: interspecific variation in origins and investment. Zoomorphology 123, 55–64. https://doi.org/10.1007/s00435-003-0094-z
Phylogeny	Soares, K.D.A., Mathubara, K., 2022. Combined phylogeny and new classification of catsharks (Chondrichthyes: Elasmobranchii: Carcharhiniformes). Zoological Journal of the Linnean Society 195, 761–814. https://doi.org/10.1093/zoolinnean/zlab108
Physiology	Söderström, V., Renshaw, G.M.C., Nilsson, G.E., 1999. Brain blood flow and blood pressure during hypoxia in the epaulette shark Hemiscyllium ocellatum, a hypoxia-tolerant elasmobranch. Journal of Experimental Biology, 202(7), 829–835.
Physiology	Speers-Roesch, B., Brauner, C.J., Farrell, A.P., Hickey, A.J.R., Renshaw, G.M.C., Wang, Y.S., Richards, J.G., 2012a. Hypoxia tolerance in elasmobranchs. II. Cardiovascular function and tissue metabolic responses during progressive and relative hypoxia exposures. Journal of Experimental Biology 215, 103–114. https://doi.org/10.1242/jeb.059667
Physiology	Speers-Roesch, B., Richards, J.G., Brauner, C.J., Farrell, A.P., Hickey, A.J.R., Wang, Y.S., Renshaw, G.M.C., 2012b. Hypoxia tolerance in elasmobranchs. I. Critical oxygen tension as a measure of blood oxygen transport during hypoxia exposure. Journal of Experimental Biology 215, 93–102. https://doi.org/10.1242/jeb.059642
Physiology	Stensløkken, K.-O., Sundin, L., Renshaw, G.M.C., Nilsson, G.E., 2004. Adenosinergic and cholinergic control mechanisms during hypoxia in the

	epaulette shark (Hemiscyllium ocellatum), with emphasis on branchial circulation. Journal of Experimental Biology 207, 4451–4461. https://doi.org/10.1242/jeb.01291
Morphology	Sternes, P.C., Shimada, K., 2020. Body forms in sharks (Chondrichthyes: Elasmobranchii) and their functional, ecological, and evolutionary implications. Zoology 140, 125799. https://doi.org/10.1016/j.zool.2020.125799
Physiology	Thomas, P.A., Peele, E.E., Wheeler, C.R., Yopak, K., Rummer, J.L., Mandelman, J.W., Kinsey, S.T., 2023. Effects of projected end-of-century temperature on the muscle development of neonate epaulette sharks, Hemiscyllium ocellatum. Mar Biol 170, 71. https://doi.org/10.1007/s00227-023-04218-z
Genomics	Torralba Sáez, M., Hofreiter, M., Straube, N., 2024. Shark genome size evolution and its relationship with cellular, life-history, ecological, and diversity traits. Scientific Reports 14, 8909. https://doi.org/10.1038/s41598-024-59202-4
Kinematics	Travis, K. G., 2020. Comparative Biomechanics of Submerged and Partially Emerged Walking in the Epaulette Shark (Hemiscyllium ocellatum). California State University, Long Beach ProQuest Dissertations & Theses. 28001935.
Conservation	VanderWright, W.J., Dudgeon, C.L., Erdmann, M.V., Sianipar, A., Dulvy, N.K., 2022. Extinction Risk and the Small Population Paradigm in the Micro-Endemic Radiation of Epaulette Sharks, in: DellaSala, D.A., Goldstein, M.I. (Eds.), Imperiled: The Encyclopedia of Conservation. Elsevier, Oxford, pp. 752–762. https://doi.org/10.1016/B978-0-12-821139-7.00130-6
Classification	Weigmann, S., 2016. Annotated checklist of the living sharks, batoids and chimaeras (Chondrichthyes) of the world, with a focus on biogeographical diversity. Journal of Fish Biology 88, 837–1037. https://doi.org/10.1111/jfb.12874
Physiology	Wells, R.M.G., Baldwin, J., Ryder, J.M., 1992. Respiratory function and nucleotide composition of erythrocytes from tropical elasmobranchs. Comparative Biochemistry and Physiology Part A: Physiology 103, 157–162. https://doi.org/10.1016/0300-9629(92)90256-P
Developmental biology	West, G.J. & Carter, S., 1990. Observations on the development and growth of the epaulette shark Hemiscyllium ocellatum (Bonnaterre) in captivity. Journal of Aquariculture and Aquatic Sciences, 4, 111–117.
Classification	Wheeler, A., 1986. Catalogue of the Natural History Drawings commissioned by Joseph Banks on the Endeavour Voyage 1768-1771 Held in the British Museum (Natural History). Bulletin of the British Museum of Natural History (Historical Series), 13, 1–172.
Physiology	Wheeler, C.R., Awruch, C.A., Mandelman, J.W., Rummer, J.L., 2025. Assessing the metabolic and physiological costs of oviparity in the epaulette shark (Hemiscyllium ocellatum). Biology Open 14, bio062076. https://doi.org/10.1242/bio.062076

Physiology	Wheeler, C.R., Irschick, D.J., Mandelman, J.W., Rummer, J.L., 2023. Nonlethally assessing elasmobranch ontogenetic shifts in energetics. Journal of Fish Biology 103, 235–246. https://doi.org/10.1111/jfb.15425
Physiology	Wheeler, C.R., Lang, B.J., Mandelman, J.W., Rummer, J.L., 2022. The upper thermal limit of epaulette sharks (Hemiscyllium ocellatum) is conserved across three life history stages, sex and body size. Conservation Physiology 10, coac074. https://doi.org/10.1093/conphys/coac074
Physiology	Wheeler, C.R., Rummer, J.L., 2024. Evidence of dystocia in an oviparous shark. Journal of Fish Biology 105, 1004–1007. https://doi.org/10.1111/jfb.15819
Physiology	Wheeler, C.R., Rummer, J.L., Bailey, B., Lockwood, J., Vance, S., Mandelman, J.W., 2021. Future thermal regimes for epaulette sharks (Hemiscyllium ocellatum): growth and metabolic performance cease to be optimal. Sci Rep 11, 454. https://doi.org/10.1038/s41598-020-79953-0
Sensory biology	Winther-Janson, M., Wueringer, B.E., Seymour, J.E., 2012. Electroreceptive and Mechanoreceptive Anatomical Specialisations in the Epaulette Shark (Hemiscyllium ocellatum). PLOS ONE 7, e49857. https://doi.org/10.1371/journal.pone.0049857
Physiology	Wise, G., Dodd, P. R., Renshaw, G. M. C., 1998. The kinetics of specific tritiated flunitrazepam binding in the brain of the epaulette shark (Hemiscyllium ocellatum).
Physiology	Wise, G., Mulvey, J.M., Renshaw, G.M.C., 1998. Hypoxia tolerance in the epaulette shark (Hemiscyllium ocellatum). J EXP ZOOL 281, 1–5. https://doi.org/10.1002/(SICI)1097-010X(19980501)281:1%253C1::AID-JEZ1%253E3.0.CO;2-S
Conservation	Wosnick, N., Leite, R.D., Giareta, E.P., Morick, D., Musyl, M., 2022. Global assessment of shark strandings. Fish and Fisheries 23, 786–799. https://doi.org/10.1111/faf.12648
Kinematics	Wu, E.H., 1994. Kinematic analysis of jaw protrusion in orectolobiform sharks: A new mechanism for jaw protrusion in elasmobranchs. Journal of Morphology 222, 175–190. https://doi.org/10.1002/jmor.1052220205
Comparative anatomy	Yopak, K.E., Lisney, T.J., 2012. Allometric Scaling of the Optic Tectum in Cartilaginous Fishes. Brain Behavior and Evolution 80, 108–126. https://doi.org/10.1159/000339875
Sensory biology	Yopak, K.E., Lisney, T.J., Collin, S.P., 2015. Not all sharks are “swimming noses”: variation in olfactory bulb size in cartilaginous fishes. Brain Structure and Function 220, 1127–1143. https://doi.org/10.1007/s00429-014-0705-0
Comparative anatomy	Yopak, K.E., Lisney, T.J., Collin, S.P., Montgomery, J.C., 2007. Variation in Brain Organization and Cerebellar Foliation in Chondrichthyans: Sharks and Holocephalans. Brain Behavior and Evolution 69, 280–300. https://doi.org/10.1159/000100037
Comparative anatomy	Yopak, K.E., Lisney, T.J., Darlington, R.B., Collin, S.P., Montgomery, J.C., Finlay, B.L., 2010. A conserved pattern of brain scaling from sharks to primates. Proceedings of the National Academy of Sciences 107, 12946–12951. https://doi.org/10.1073/pnas.1002195107

Figure 2

Bouyoucos, I.A., 2021. Walking sharks cannot beat the heat. Conservation Physiology 9, coab035. https://doi.org/10.1093/conphys/coab035

Chapman, C. A., 2009. Anoxia Survival Strategies in the Grey Carpet Shark (Chiloscyllium punctatum) and the Epaulette Shark (Hemiscyllium ocellatum). PhD Thesis, School of Physiotherapy and Exercise Science, Griffith University. https://doi.org/10.25904/1912/594.

Chapman, C.A., Harahush, B.K., Renshaw, G.M.C., 2011. The physiological tolerance of the grey carpet shark (Chiloscyllium punctatum) and the epaulette shark (Hemiscyllium ocellatum) to anoxic exposure at three seasonal temperatures. Fish Physiol Biochem 37, 387–399. https://doi.org/10.1007/s10695-010-9439-y

Chapman, C.A., Renshaw, G.M.C., 2009. Hematological responses of the grey carpet shark (Chiloscyllium punctatum) and the epaulette shark (Hemiscyllium ocellatum) to anoxia and re-oxygenation. Journal of Experimental Zoology Part A: Ecological Genetics and Physiology 311A, 422–438. https://doi.org/10.1002/jez.539

Devaux, J.B.L., Hickey, A.J.R., Renshaw, G.M.C., 2023. Succinate-mediated reactive oxygen species production in the anoxia-tolerant epaulette (Hemiscyllium ocellatum) and grey carpet (Chiloscyllium punctatum) sharks. Biology Letters 19, 20230344. https://doi.org/10.1098/rsbl.2023.0344

Devaux, J.B.L., Hickey, A.J.R., Renshaw, G.M.C., 2019. Mitochondrial plasticity in the cerebellum of two anoxia-tolerant sharks: contrasting responses to anoxia/re-oxygenation. Journal of Experimental Biology 222, jeb191353. https://doi.org/10.1242/jeb.191353

Dowd, W.W., Renshaw, G.M.C., Cech, J.J., Kültz, D., 2010. Compensatory proteome adjustments imply tissue-specific structural and metabolic reorganization following episodic hypoxia or anoxia in the epaulette shark (Hemiscyllium ocellatum). Physiological Genomics 42, 93–114. https://doi.org/10.1152/physiolgenomics.00176.2009

Gervais, C.R., Nay, T.J., Renshaw, G., Johansen, J.L., Steffensen, J.F., Rummer, J.L., 2018. Too hot to handle? Using movement to alleviate effects of elevated temperatures in a benthic elasmobranch, Hemiscyllium ocellatum. Mar Biol 165, 162. https://doi.org/10.1007/s00227-018-3427-7

Heinrich, D.D.U., Rummer, J.L., Morash, A.J., Watson, S.-A., Simpfendorfer, C.A., Heupel, M.R., Munday, P.L., 2014. A product of its environment: the epaulette shark (Hemiscyllium ocellatum) exhibits physiological tolerance to elevated environmental CO(2). Conservation Physiology 2, cou047. https://doi.org/10.1093/conphys/cou047

Heinrich, D.D.U., Watson, S.-A., Rummer, J.L., Brandl, S.J., Simpfendorfer, C.A., Heupel, M.R., Munday, P.L., 2016. Foraging behaviour of the epaulette shark Hemiscyllium ocellatum is not affected by elevated CO2. ICES Journal of Marine Science 73, 633–640. https://doi.org/10.1093/icesjms/fsv085

Hickey, A.J.R., Renshaw, G.M.C., Speers-Roesch, B., Richards, J.G., Wang, Y., Farrell, A.P., Brauner, C.J., 2012. A radical approach to beating hypoxia: depressed free radical release from heart fibres of the hypoxia-tolerant epaulette shark (Hemiscyllum ocellatum). J Comp Physiol B 182, 91–100. https://doi.org/10.1007/s00360-011-0599-6

Leveelahti, L., Rytkönen, K.T., Renshaw, G.M.C., Nikinmaa, M., 2014. Revisiting redox-active antioxidant defenses in response to hypoxic challenge in both hypoxia-tolerant and hypoxia-sensitive fish species. Fish Physiology and Biochemistry 40, 183–191. https://doi.org/10.1007/s10695-013-9835-1

Mulvey, J.M., Renshaw, G.M.C., 2009. GABA is not elevated during neuroprotective neuronal depression in the hypoxic epaulette shark (Hemiscyllium ocellatum), in: Comparative Biochemistry and Physiology Part A: Molecular & Integrative Physiology. pp. 273–277. https://doi.org/10.1016/j.cbpa.2008.10.017

Mulvey, J.M., Renshaw, G.M.C., 2000. Neuronal oxidative hypometabolism in the brainstem of the epaulette shark (*Hemiscyllium ocellatum)* in response to hypoxic pre-conditioning. Neuroscience Letters 290, 1–4. https://doi.org/10.1016/S0304-3940(00)01321-5

Nay, T.J., Longbottom, R.J., Gervais, C.R., Johansen, J.L., Steffensen, J.F., Rummer, J.L., Hoey, A.S., 2021. Regulate or tolerate: Thermal strategy of a coral reef flat resident, the epaulette shark, Hemiscyllium ocellatum. Journal of Fish Biology 98, 723–732. https://doi.org/10.1111/jfb.14616

Nilsson, G.E., Renshaw, G.M.C., 2004. Hypoxic survival strategies in two fishes: Extreme anoxia tolerance in the North European crucian carp and natural hypoxic preconditioning in a coral-reef shark. J EXP BIOL 207, 3131–3139. https://doi.org/10.1242/jeb.00979

Renshaw, G.M.C., Neuzil, J., Girjes, A., 2004. Changes in gene expression in response to anoxic preconditioning. The Molecular Basis, 13.

Renshaw, G.M.C., Kerrisk, C.B., Nilsson, G.E., 2002. The role of adenosine in the anoxic survival of the epaulette shark, *Hemiscyllium ocellatum*. Comparative Biochemistry and Physiology Part B: Biochemistry and Molecular Biology 131, 133–141. https://doi.org/10.1016/S1096-4959(01)00484-5

Renshaw, G.M.C., Wise, G., Dodd, P.R., 2010. Ecophysiology of neuronal metabolism in transiently oxygen-depleted environments: Evidence that GABA is accumulated pre-synaptically in the cerebellum. Comparative Biochemistry and Physiology Part A: Molecular & Integrative Physiology 155, 486–492. https://doi.org/10.1016/j.cbpa.2009.10.039

Routley, M.H., Nilsson, G.E., Renshaw, G.M.C., 2002. Exposure to hypoxia primes the respiratory and metabolic responses of the epaulette shark to progressive hypoxia. Comparative Biochemistry and Physiology Part A: Molecular & Integrative Physiology 131, 313–321. https://doi.org/10.1016/S1095-6433(01)00484-6

Schwieterman, G.D., Rummer, J.L., Bouyoucos, I.A., Bushnell, P.G., Brill, R.W., 2021. A lack of red blood cell swelling in five elasmobranch fishes following air exposure and exhaustive exercise. Comparative Biochemistry and Physiology Part A: Molecular & Integrative Physiology 258, 110978. https://doi.org/10.1016/j.cbpa.2021.110978

Söderström, V., Renshaw, G.M.C., Nilsson, G.E., 1999. Brain blood flow and blood pressure during hypoxia in the epaulette shark Hemiscyllium ocellatum, a hypoxia-tolerant elasmobranch. Journal of Experimental Biology, 202(7), 829–835.

Stensløkken, K.-O., Sundin, L., Renshaw, G.M.C., Nilsson, G.E., 2004. Adenosinergic and cholinergic control mechanisms during hypoxia in the epaulette shark (Hemiscyllium ocellatum), with emphasis on branchial circulation. Journal of Experimental Biology 207, 4451–4461. https://doi.org/10.1242/jeb.01291

Thomas, P.A., Peele, E.E., Wheeler, C.R., Yopak, K., Rummer, J.L., Mandelman, J.W., Kinsey, S.T., 2023. Effects of projected end-of-century temperature on the muscle development of neonate epaulette sharks, Hemiscyllium ocellatum. Mar Biol 170, 71. https://doi.org/10.1007/s00227-023-04218-z

Wheeler, C.R., Lang, B.J., Mandelman, J.W., Rummer, J.L., 2022. The upper thermal limit of epaulette sharks (Hemiscyllium ocellatum) is conserved across three life history stages, sex and body size. Conservation Physiology 10, coac074. https://doi.org/10.1093/conphys/coac074

Wheeler, C.R., Rummer, J.L., Bailey, B., Lockwood, J., Vance, S., Mandelman, J.W., 2021. Future thermal regimes for epaulette sharks (Hemiscyllium ocellatum): growth and metabolic performance cease to be optimal. Sci Rep 11, 454. https://doi.org/10.1038/s41598-020-79953-0
Wise, G., Dodd, P. R., Renshaw, G. M. C., 1998. The kinetics of specific tritiated flunitrazepam binding in the brain of the epaulette shark (Hemiscyllium ocellatum).
Wise, G., Mulvey, J.M., Renshaw, G.M.C., 1998. Hypoxia tolerance in the epaulette shark (Hemiscyllium ocellatum). J EXP ZOOL 281, 1–5. https://doi.org/10.1002/(SICI)1097-010X(19980501)281:1%253C1::AID-JEZ1%253E3.0.CO;2-S

Supplemental table 2: sources used in Figure 2. Sources were compiled from publications presenting original or synthesized information on the *Hemiscyllium ocellatum*, identified primarily through Shark References and supplemented with Google Scholar search results. Publications that did not contribute original or synthesized data and did not use *H. ocellatum* as a study species were excluded.

Category	Stressor	Reference
Physiology	Temperature	Bouyoucos, I.A., 2021. Walking sharks cannot beat the heat. Conservation Physiology 9, coab035. https://doi.org/10.1093/conphys/coab035
Physiology	Hypoxia	Chapman, C. A., 2009. Anoxia Survival Strategies in the Grey Carpet Shark (Chiloscyllium punctatum) and the Epaulette Shark (Hemiscyllium ocellatum). PhD Thesis, School of Physiotherapy and Exercise Science, Griffith University. https://doi.org/10.25904/1912/594.
Physiology	Hypoxia	Chapman, C.A., Renshaw, G.M.C., 2009. Hematological responses of the grey carpet shark (Chiloscyllium punctatum) and the epaulette shark (Hemiscyllium ocellatum) to anoxia and re-oxygenation. Journal of Experimental Zoology Part A: Ecological Genetics and Physiology 311A, 422–438. https://doi.org/10.1002/jez.539
Physiology	Hypoxia	Chapman, C.A., Harahush, B.K., Renshaw, G.M.C., 2011. The physiological tolerance of the grey carpet shark (Chiloscyllium punctatum) and the epaulette shark (Hemiscyllium ocellatum) to anoxic exposure at three seasonal temperatures. Fish Physiol Biochem 37, 387–399. https://doi.org/10.1007/s10695-010-9439-y
Phylogeny	Hypoxia	Devaux, J.B.L., Hickey, A.J.R., Renshaw, G.M.C., 2019. Mitochondrial plasticity in the cerebellum of two anoxia-tolerant sharks: contrasting responses to anoxia/re-oxygenation. Journal of Experimental Biology 222, jeb191353. https://doi.org/10.1242/jeb.191353
Phylogeny	Hypoxia	Devaux, J.B.L., Hickey, A.J.R., Renshaw, G.M.C., 2023. Succinate-mediated reactive oxygen species production in the anoxia-tolerant epaulette (Hemiscyllium ocellatum) and grey carpet (Chiloscyllium punctatum) sharks. Biology Letters 19, 20230344. https://doi.org/10.1098/rsbl.2023.0344
Physiology	Hypoxia	Dowd, W.W., Renshaw, G.M.C., Cech, J.J., Kültz, D., 2010. Compensatory proteome adjustments imply tissue-specific structural and metabolic reorganization following episodic hypoxia or anoxia in the epaulette shark (Hemiscyllium ocellatum). Physiological Genomics 42, 93–114. https://doi.org/10.1152/physiolgenomics.00176.2009
Physiology	Temperature	Gervais, C.R., Nay, T.J., Renshaw, G., Johansen, J.L., Steffensen, J.F., Rummer, J.L., 2018. Too hot to handle? Using movement to alleviate effects of elevated temperatures in a benthic

		elasmobranch, Hemiscyllium ocellatum. Mar Biol 165, 162. https://doi.org/10.1007/s00227-018-3427-7
Physiology	CO2	Heinrich, D.D.U., Rummer, J.L., Morash, A.J., Watson, S.-A., Simpfendorfer, C.A., Heupel, M.R., Munday, P.L., 2014. A product of its environment: the epaulette shark (Hemiscyllium ocellatum) exhibits physiological tolerance to elevated environmental CO(2). Conservation Physiology 2, cou047. https://doi.org/10.1093/conphys/cou047
Physiology	CO2	Heinrich, D.D.U., Watson, S.-A., Rummer, J.L., Brandl, S.J., Simpfendorfer, C.A., Heupel, M.R., Munday, P.L., 2016. Foraging behaviour of the epaulette shark Hemiscyllium ocellatum is not affected by elevated CO2. ICES Journal of Marine Science 73, 633–640. https://doi.org/10.1093/icesjms/fsv085
Physiology	Hypoxia	Hickey, A.J.R., Renshaw, G.M.C., Speers-Roesch, B., Richards, J.G., Wang, Y., Farrell, A.P., Brauner, C.J., 2012. A radical approach to beating hypoxia: depressed free radical release from heart fibres of the hypoxia-tolerant epaulette shark (Hemiscyllum ocellatum). J Comp Physiol B 182, 91–100. https://doi.org/10.1007/s00360-011-0599-6
Physiology	Hypoxia	Leveelahti, L., Rytkönen, K.T., Renshaw, G.M.C., Nikinmaa, M., 2014. Revisiting redox-active antioxidant defenses in response to hypoxic challenge in both hypoxia-tolerant and hypoxia-sensitive fish species. Fish Physiology and Biochemistry 40, 183–191. https://doi.org/10.1007/s10695-013-9835-1
Physiology	Hypoxia	Mulvey, J.M., Renshaw, G.M.C., 2009. GABA is not elevated during neuroprotective neuronal depression in the hypoxic epaulette shark (Hemiscyllium ocellatum), in: Comparative Biochemistry and Physiology Part A: Molecular & Integrative Physiology. pp. 273–277. https://doi.org/10.1016/j.cbpa.2008.10.017
Physiology	Hypoxia	Mulvey, J.M., Renshaw, G.M.C., 2000. Neuronal oxidative hypometabolism in the brainstem of the epaulette shark (Hemiscyllium ocellatum) in response to hypoxic pre-conditioning. Neuroscience Letters 290, 1–4. https://doi.org/10.1016/S0304-3940(00)01321-5
Physiology	Temperature	Nay, T.J., Longbottom, R.J., Gervais, C.R., Johansen, J.L., Steffensen, J.F., Rummer, J.L., Hoey, A.S., 2021. Regulate or tolerate: Thermal strategy of a coral reef flat resident, the epaulette shark, Hemiscyllium ocellatum. Journal of Fish Biology 98, 723–732. https://doi.org/10.1111/jfb.14616
Physiology	Hypoxia	Renshaw, G.M.C., Neuzil, J., Girjes, A., 2004. Changes in gene expression in response to anoxic preconditioning. The Molecular Basis, 13.
Physiology	Hypoxia	Renshaw, G.M.C., Kerrisk, C.B., Nilsson, G.E., 2002. The role of adenosine in the anoxic survival of the epaulette shark,

		Hemiscyllium ocellatum. Comparative Biochemistry and Physiology Part B: Biochemistry and Molecular Biology 131, 133–141. https://doi.org/10.1016/S1096-4959(01)00484-5
Physiology	Hypoxia	Renshaw, G.M.C., Wise, G., Dodd, P.R., 2010. Ecophysiology of neuronal metabolism in transiently oxygen-depleted environments: Evidence that GABA is accumulated pre-synaptically in the cerebellum. Comparative Biochemistry and Physiology Part A: Molecular & Integrative Physiology 155, 486–492. https://doi.org/10.1016/j.cbpa.2009.10.039
Physiology	Hypoxia	Routley, M.H., Nilsson, G.E., Renshaw, G.M.C., 2002. Exposure to hypoxia primes the respiratory and metabolic responses of the epaulette shark to progressive hypoxia. Comparative Biochemistry and Physiology Part A: Molecular & Integrative Physiology 131, 313–321. https://doi.org/10.1016/S1095-6433(01)00484-6
Physiology	Hypoxia	Schwieterman, G.D., Rummer, J.L., Bouyoucos, I.A., Bushnell, P.G., Brill, R.W., 2021. A lack of red blood cell swelling in five elasmobranch fishes following air exposure and exhaustive exercise. Comparative Biochemistry and Physiology Part A: Molecular & Integrative Physiology 258, 110978. https://doi.org/10.1016/j.cbpa.2021.110978
Physiology	Hypoxia	Söderström, V., Renshaw, G.M.C., Nilsson, G.E., 1999. Brain blood flow and blood pressure during hypoxia in the epaulette shark Hemiscyllium ocellatum, a hypoxia-tolerant elasmobranch. Journal of Experimental Biology, 202(7), 829–835.
Physiology	Hypoxia	Stensløkken, K.-O., Sundin, L., Renshaw, G.M.C., Nilsson, G.E., 2004. Adenosinergic and cholinergic control mechanisms during hypoxia in the epaulette shark (Hemiscyllium ocellatum), with emphasis on branchial circulation. Journal of Experimental Biology 207, 4451–4461. https://doi.org/10.1242/jeb.01291
Physiology	Temperature	Thomas, P.A., Peele, E.E., Wheeler, C.R., Yopak, K., Rummer, J.L., Mandelman, J.W., Kinsey, S.T., 2023. Effects of projected end-of-century temperature on the muscle development of neonate epaulette sharks, Hemiscyllium ocellatum. Mar Biol 170, 71. https://doi.org/10.1007/s00227-023-04218-z
Physiology	Temperature	Wheeler, C.R., Rummer, J.L., Bailey, B., Lockwood, J., Vance, S., Mandelman, J.W., 2021. Future thermal regimes for epaulette sharks (Hemiscyllium ocellatum): growth and metabolic performance cease to be optimal. Sci Rep 11, 454. https://doi.org/10.1038/s41598-020-79953-0
Physiology	Temperature	Wheeler, C.R., Lang, B.J., Mandelman, J.W., Rummer, J.L., 2022. The upper thermal limit of epaulette sharks (Hemiscyllium ocellatum) is conserved across three life history stages, sex and body size. Conservation Physiology 10, coac074. https://doi.org/10.1093/conphys/coac074

Physiology	Hypoxia	Wise, G., Dodd, P. R., Renshaw, G. M. C., 1998. The kinetics of specific tritiated flunitrazepam binding in the brain of the epaulette shark (Hemiscyllium ocellatum).
Physiology	Hypoxia	Wise, G., Mulvey, J.M., Renshaw, G.M.C., 1998. Hypoxia tolerance in the epaulette shark (Hemiscyllium ocellatum). J EXP ZOOL 281, 1–5. https://doi.org/10.1002/(SICI)1097-010X(19980501)281:1%253C1::AID-JEZ1%253E3.0.CO;2-S
